# [2 + 2] Photocyclization converts thermally induced spin crossover effect into “hidden hysteresis” one†

**DOI:** 10.1039/d4sc05587j

**Published:** 2025-03-25

**Authors:** Marcin Kaźmierczak, Marek Weselski, Miłosz Siczek, Juliusz A. Wolny, Volker Schünemann, Robert Bronisz

**Affiliations:** a Faculty of Chemistry, University of Wrocław F. Joliot-Curie 14, 50-383 Wrocław Poland robert.bronisz@uwr.edu.pl; b Faculty of Physics, RPTU Kaiserslautern-Landau Erwin Schrödinger Str. 46 67663 Kaiserlautern Germany

## Abstract

The light induced [2 + 2] cyclization of the flexible coumarin-based ligand (L) converts the spin crossover active HS1 ⇆ LS1 mononuclear system [Fe(L)_6_](BF_4_)_2_·4CH_3_CN (1) into the high spin 1D coordination polymer (2). The contribution of the resulting high spin form HS2 is directly related to the degree of photoconversion and, at the same time, practically does not affect the properties of the remaining thermally active spin crossover centers (HS1). The origin of such a fundamental change in properties is an appearance of strain caused by ligand dimerization, which acts directly on the metal chromophores and is transmitted to the crystal lattice. The spin state of 2 can be changed by applying pressure as well as by light irradiation revealing a “hidden hysteresis” phenomenon (*Appl. Phys. Lett.*, 2008, **93**, 21906), referring to the appearance of the low spin state not accessible through thermal activation but through reversed-LIESST. A unique feature of 2 is the feasibility to attain any steady state within the hidden hysteresis region by combination of perturbations triggered by changes in temperature and light (808 nm HS2 → LS2 and 532 nm LS2 → HS2). Such states are stable within a time scale of several hours.

## Abstract 2

Ligand photoconversion in an Fe(ii) SCO complex results in a hidden low spin state effect. Photoswitching combined with a change in temperature allows reaching any macroscopic magnetically steady state within the hidden hysteresis region.

## Graphical abstract

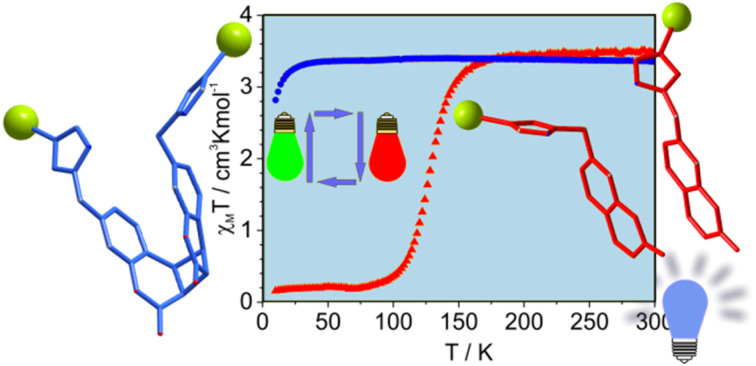

## Introduction

Intramolecular isomerization processes, occurring, for example, in bisthienylethene^1^ and azobenzene derivatives^2^ as well as involving intermolecular photoinduced [2 + 2] cyclization,^3^ are the subject of intensive research aimed at constructing building blocks of dynamically controllable structures. In solutions, [2 + 2] cyclization is generally not stereoselective.^4^ However, creating conditions conducive to the preorganization of molecules can increase control over stereoselectivity, in effect, resulting in the formation of one product instead of a mixture of isomeric species.^5^ The occurrence of the process requires a proper, relative reorientation of the molecules or their fragments, and compared to solutions, the mutual movement of molecules in the solid phase or in the gel^6^ is drastically limited, thus reducing the chances of the reaction occurring. Designing the structure in such a way that the topochemical criteria required for photocyclization will be met in the solid^7^ is difficult because, in the case of newly designed systems, especially coordination compounds, it is practically impossible to predict the packing of molecules in the crystal lattice. Nevertheless, the use of crystal engineering tools allows for more rational control over the mutual arrangement of photoreactive fragments. Intermolecular interactions,^8^ including those as specific as Ag⋯Ag^9–12^ and much less frequently Ag⋯π,^12^ Au⋯Au^13^ or halogen interactions, are used.^14^

Although the use of crystal engineering tools allows us to increase the probability of achieving the required topochemical conditions, the photocyclization process may still result in diverse yet unpredictable structures. Photodimerization in coordination compounds with a discrete structure can lead to a change in nuclearity, such as mononuclear → dinuclear^15^ or dinuclear → tetranuclear.^16^ Discrete systems can also be converted to coordination polymers.^8,9,11^ Moreover, photoconversion of coordination polymers^3,17^ can lead to a change in dimensionality: 1D → 2D,^18^ 1D → 3D^19^ or to the formation of complex systems such as polyrotaxanes.^20^ It can result in systems with interesting structural features, entailing the occurrence of host–guest^21^ interactions or characterized by permanent porosity.^22^

**Fig. 1:**
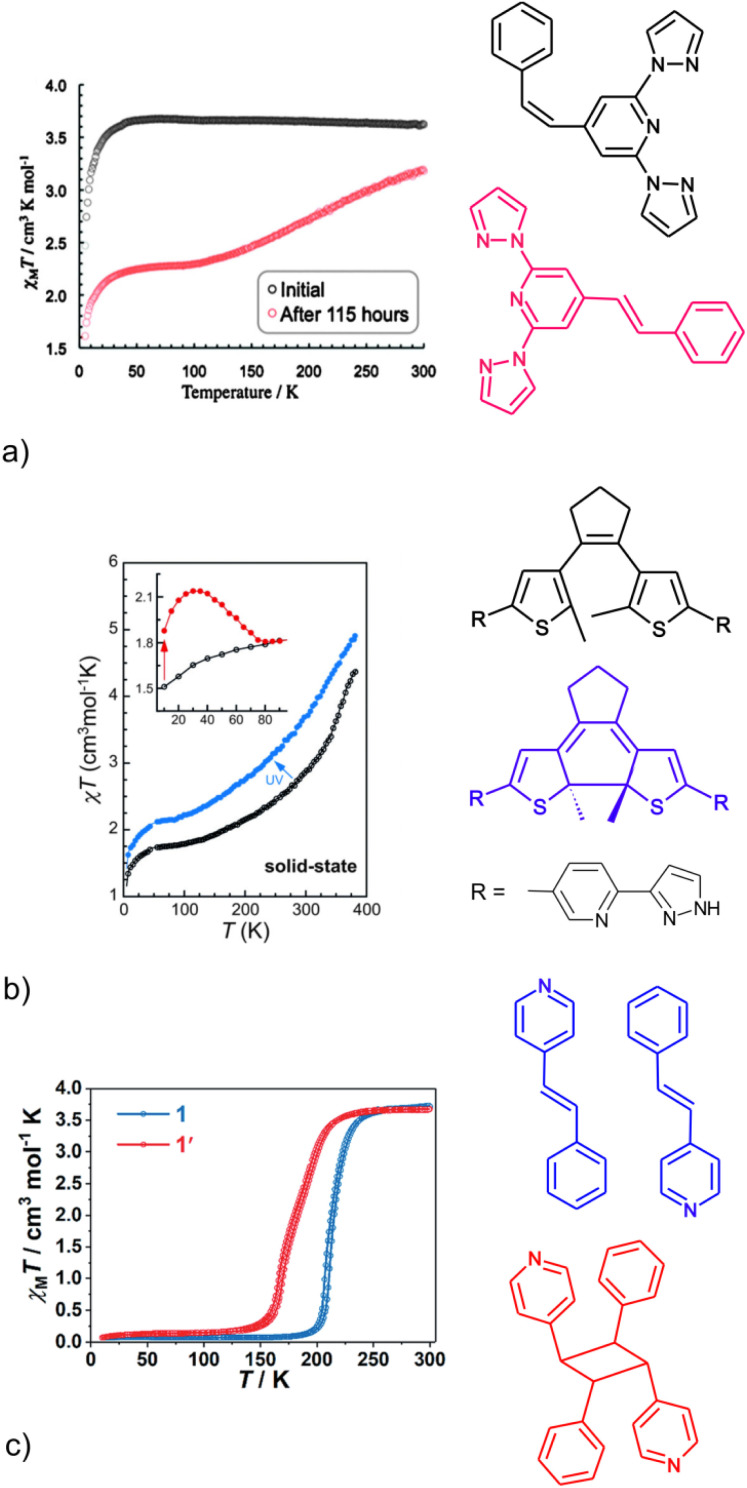
The effect of *Z*–*E* isomerization of a 4-styrylpyridine based ligand (a), ring closure-opening in a dithienylethene derivative (b) and [2 + 2] cycloaddition of 4-styrylpyridine derivatives (c) on the spin crossover properties of Fe(ii) systems. These figures were taken from ref. 24 (a), 29 (b) and 30 (c). On the right side, the structures of ligands (before and after photoconversion) are shown, with the colors corresponding to the temperature dependencies shown on the left side.

A phenomenon in which the structure of a material plays a crucial role can be observed in spin crossover coordination compounds.^23^ Concomitantly it should be highlighted that the spin crossover properties are very sensitive to structural alterations occurring in the first and further coordination spheres. Hence, there is an interest in photoswitchable systems as sources of structural disturbance. The reported compounds are few and the challenge lies in obtaining a photoresponsive system, particularly one that exhibits strong synergy between the spin state and the functional state, acting as a molecular switch. One of the most spectacular examples is the photoisomerization of *Z*-2,6-di(1*H*-pyrazol-1-yl)-4-styrylpyridine (*Z*-1) in the Fe(ii) coordination compound [Fe(*Z*-1)_2_](BF_4_)_2_ (*Z*-2).^24^ In the solid phase, *Z*-2 can be photoconverted at 420 nm to the *E*-2 isomer, and 30% of *E*-2 molecules undergo thermally induced spin crossover (Fig. 1a). Studies of azopyridine-based coordination compounds have been conducted extensively for L–B films and dispersions in a cellulose matrix, resulting in partial photoisomerization.^25^

An interesting result was obtained using a condensed system composed of 1,10-phenanthroline and 2,5-dimethylthiophene rings.^26^ This approach, based on the photoisomerization related to the formation of a linkage between two proximal thiophene rings on the outer rim of the ligand, suffers due to the lack of control over the mutual orientation of the rings in the solid. In the solid state, photoisomerization does not occur for the parallel orientation, although it occurs in solution.^27^ In contrast, with the desired anti-parallel orientation of the thiophene rings, successful switching to a spin crossover active system was observed, for which 30% of Fe(ii) ions showed a change in the spin state in the solid phase.^28^ What is significant is that the use of rigid bridging ligand molecules, leading to dimeric systems, reduced the ability to undergo photoisomerization in the solid phase (Fig. 1b).^29^ The low efficiency of photoisomerization and the gradual course of the spin crossover seem to be common features of systems based on rigid photoreactive ligand molecules. The importance of the freedom of reorientation of structural elements is emphasized by the complete transformation of the Hofmann-type network [Fe(L)_2_{Ag(CN)_2_}_2_] (L = 4-styrylpyridine or *trans*-1-(2-pyridyl)-2-(4-pyridyl)ethene) (Fig. 1c).^30^ The conversion takes place with the involvement of a guest molecule, thus increasing the ability to reorient in the crystal lattice, and it results in a change in spin crossover properties.

**Scheme 1:**
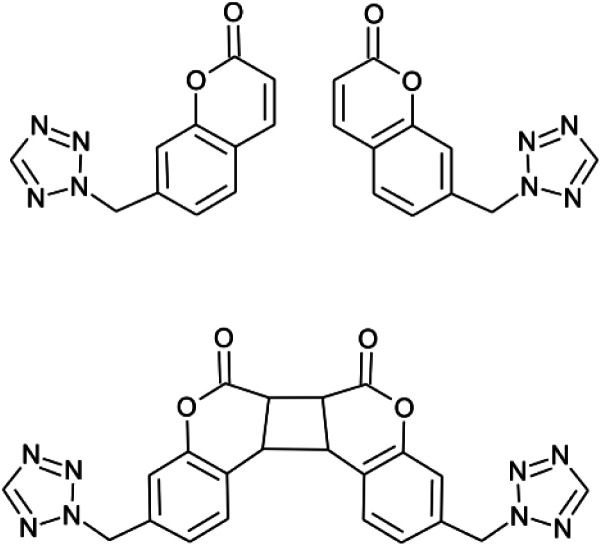
Molecular structure of 7-(tetrazol-2-ylmethyl)coumarin (L, at the top) and the product of [2 + 2] photocyclization (at the bottom).

Taking the above into account, we believe that the limitation responsible for the insignificant effect of photoconversion on spin crossover or even for photoconversion incompleteness is the rigidity of the photoreactive building blocks. Therefore we decided to design a ligand system having flexible character rather than one based on coupled or fused fragments. This approach should fulfill topochemical requirements and facilitate the adoption of orientation during photocyclization, ultimately resulting in completeness of the photoconversion process. This report presents a mononuclear spin crossover system featuring the presence of a coumarin fragment (Scheme 1) capable of photoinduced [2 + 2] cyclization. The photoconversion results in a drastic change in magnetic properties due to the reconstruction of the crystal structure from discrete to a one dimensional coordination polymer. It also involves a transition from the spin crossover active compound to a HS species exhibiting the “hidden hysteresis”^31^ effect, that offers extraordinary possibilities to alter the magnetic properties within the hysteresis loop.

## Results and discussion

### General strategy for photoreactive ligand structure selection

In order to obtain a photoswitchable system we designed a molecule which contains photoreactive fragments on the periphery of the octahedral structure. This should provide the possibility of intermolecular dimerization in all directions of the lattice. At the same time, however, the topochemical requirements necessitate a proper matching of photoreactive fragments to each other. This is crucially determined by the structure of the ligand molecule and its adaptability combined with the formation of intermolecular contacts. As a result, we synthesized a ligand that combines a monodentally coordinating 2-substituted tetrazole ring, responsible for incorporating the spin crossover function in connection with Fe(ii) ions,^32^ with a coumarin fragment capable of forming layered structures in the crystal lattice. An important structural element providing the molecule with adaptive abilities was the methylene linker, which gave it flexibility, and thus facilitated the ability of photoreactive fragments to obtain the required relative orientation. 7-(Tetrazol-2-ylmethyl)coumarin (L) was prepared by the reaction of 7-(bromomethyl)coumarin with tetrazole (see the ESI† for synthetic details) and isolated with 22% yield as colorless stable crystals, soluble in common solvents such as acetonitrile or methanol.

### Synthesis, structure and characterization of the spin crossover properties of compound 1

A successful formation of the coordination compound [Fe(L)_6_](BF_4_)_2_·4CH_3_CN (1) was accomplished in acetonitrile by the reaction between L and iron(ii) tetrafluoroborate carried out at a molar ratio of 6 : 1 (ESI†). The complex crystallizes as colorless crystals, stable during storage in air. The sample intended for further studies was stored in the dark.

Thermogravimetric analysis revealed that only heating above 373 K is accompanied by mass loss which corresponds to removal of four non-coordinating acetonitrile molecules (Fig. S1†). Cooling the crystalline sample of 1 causes the appearance of a purple color, which indicates formation of the LS form of compound 1.

**Fig. 2:**
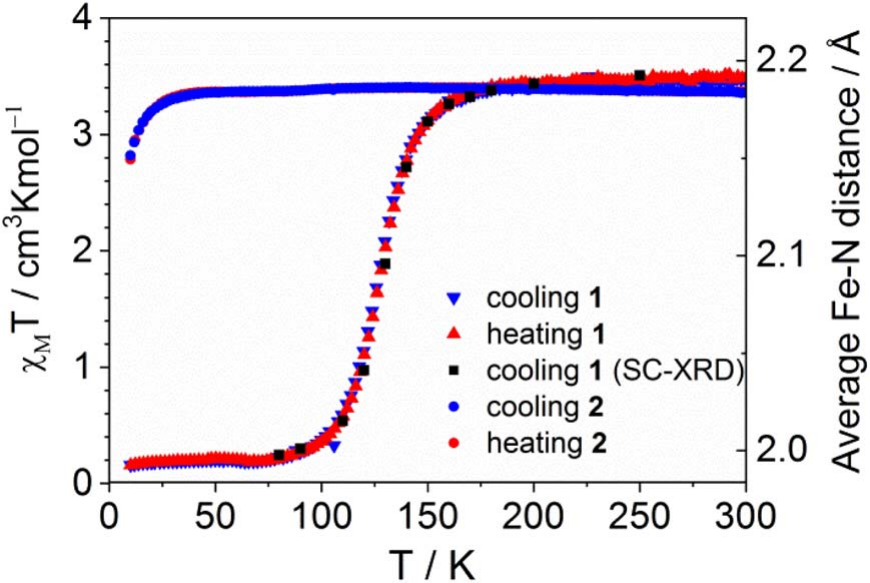
*χ*_M_ *T*(*T*) dependences for 1 (filled triangles) and 2 (filled circles) recorded in cooling (blue) and heating (red) modes. Black squares denote average Fe–N distances determined during cooling. Temperature scan rate: 1 K min^−1^. Applied magnetic field: 1 T.

At 295 K *χ*_M_*T* for 1 is equal to 3.48 cm^3^ K mol^−1^ which is characteristic for the high spin (HS) form of octahedral Fe(ii) coordination compounds. 1 remains in its HS form down to 180 K (Fig. 2). Further cooling involves an abrupt and complete spin crossover (*T*_1/2_ = 130 K). At 100 K the value of *χ*_M_*T* is equal to 0.10 cm^3^ K mol^−1^. After cooling to 10 K the measurement in the heating mode was carried out. The *χ*_M_*T*(*T*) dependence is practically the same as that recorded during cooling. Any further cooling/heating cycles do not affect the *χ*_M_*T*(*T*) dependence. Rapid cooling of sample 1 at 10 K does not involve freezing the HS form. Measurements performed for several samples obtained from different synthetic batches lead to the same *χ*_M_*T*(*T*) dependences indicating reproducibility of the reported properties.

Irradiation of 1 at 10 K with light of a wavelength of 532 nm induces low spin (LS1) to high spin (HS1*) switching (Fig. S2†). The generated high spin phase is metastable (HS1*) and immediately after switching off the light, relaxation to the LS1 phase occurs (Fig. S3–S5, see the ESI† for details). Continuous irradiation, accompanied by an elevation of temperature (0.3 K min^−1^) increases the temperature range of stability of the metastable high spin form. The HS1* → LS1 relaxation begins above 40 K. After reaching 80 K, the measurement under light irradiation was continued in cooling mode, revealing the occurrence of light induced thermal hysteresis (LITH, Fig. S2†).

**Fig. 3:**
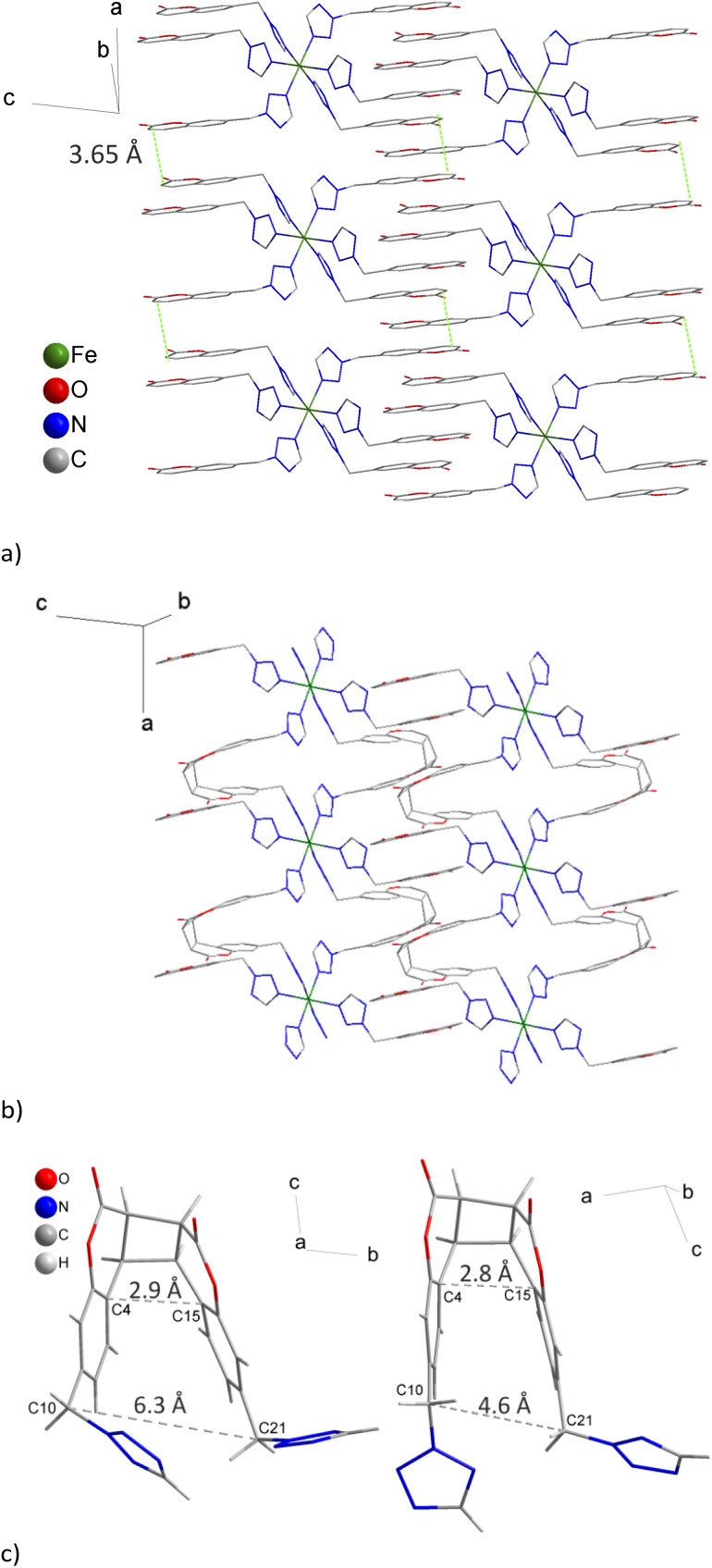
The complex cations in 1 ((a) 250 K) and the arrangement of polymeric chains along the *a* direction in 2 ((b) 250 K) as a result of [2 + 2] photocyclization. Green lines (a) connect the coumarin fragments involved in photocyclization. Hydrogen atoms, anions and acetonitrile molecules were omitted for clarity. Cyclized non-coordinated (left) and coordinated (right) forms of ligands are presented in (c).

The coordination compound 1 crystallizes in the *P*1̄ space group (Table S1†). There is one crystallographically independent Fe(ii) ion in the crystal lattice. It is coordinated with six ligand molecules, coordinating monodentally through the *exo* located nitrogen atoms of the tetrazole rings (Fig. 3a). The coordination geometry is slightly elongated octahedron with a set of four donor atoms at a distance of 2.183(2) for N12 and 2.187(2) Å for N8 at 250 K. Fe–N4 bond lengths for axially positioned donor atoms are equal to 2.208(2) Å. Thus Fe–N distances at 250 K are typical for the high spin (HS) form. There are three crystallographically independent ligand molecules and each of them adopts different conformations (Table S2†). This is the result of the presence of a methylene linker tethering the coumarin fragment to the tetrazole ring. Therefore, the flexibility of ligand molecules results in the orientation of the coumarin fragments within common planes in the complex cation. Hence, packing of the complex cations in the crystal lattice leads to formation of columns (Fig. 3a and S6†). There are established intermolecular contacts between coumarin fragments from neighboring columns (Fig. S7a†). While the tetrafluoroborate anions are disordered and acetonitrile molecules are organized, both participate in the formation of a network of intermolecular interactions (Table S3†).

Spin crossover in 1 is accompanied by the shortening of Fe–N distances (Table S2†) and the temperature dependence remains in agreement with the results of magnetic studies (Fig. 2). At 80 K, Fe–N bond lengths are equal to 1.984(1), 1.993(1) and 2.016(1) Å, indicating a shortening of approximately 0.19 Å, which corresponds to complete spin crossover and is consistent with the results of magnetic and Mössbauer spectroscopy studies.

The N–Fe–N angles in 1 range from 87.58(5) to 92.42(5)° (*Σ* = 15.0°), which are comparable to those found for the high spin form at 250 K, which adopts values from 87.18(6) to 92.82(6)° (*Σ* = 15.3°; *Σ* denotes the sum of the twelve *cis*-N–Fe–N angles). The spin state switching in 1 does not involve changes in the conformation of the ligand molecules. Orientations of counterions and noncoordinated acetonitrile molecules remain unchanged (Fig. S7a†). In effect, the network of intermolecular interactions remains practically unaffected by spin crossover (Table S3†).

### Photoconversion of 1 → 2

The inspection of the relative positions of coumarin fragments indicated the occurrence of two kinds of arrangements between nearest neighbors in the crystal lattice of 1. The distance between parallelly oriented coumarin moieties coming from the same [FeL_6_]^2+^ complex cation is above 7 Å which excludes the possibility of photocyclization. Nevertheless, the shortest distance between centers of double bonds of coumarin moieties from neighboring complex cations creating columns (along the *a* direction) equals *ca.* 3.65 Å (Fig. 3a). These coumarin fragments are arranged head-to-head in a *cis* manner.

**Fig. 4:**
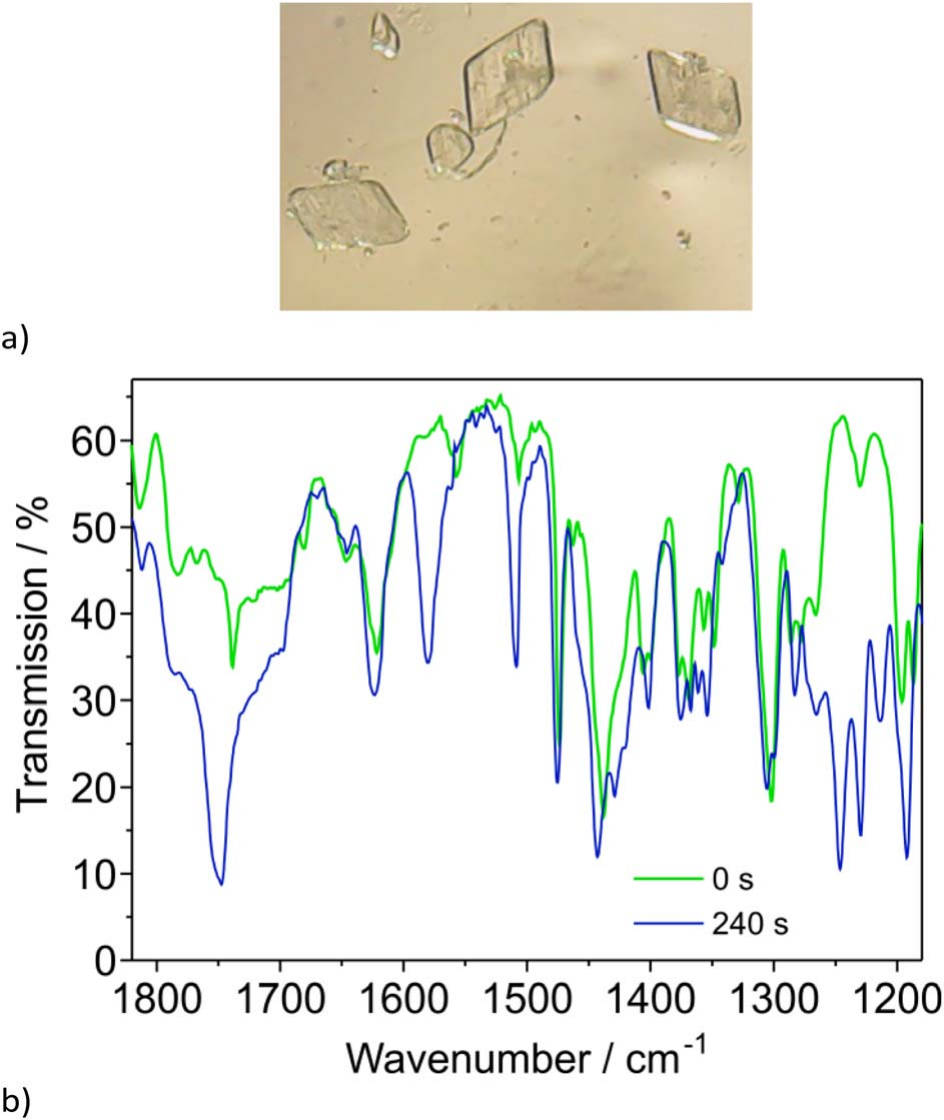
Picture of crystals of 1 (a) and FTIR microscopy spectra (b) as a function of time of irradiation (365 nm, 295 K).

FTIR microscopy experiments conducted at 295 K on very thin crystals of 1 (Fig. 4a) revealed that photocyclization occurs under light of wavelength 365 nm (Fig. 4b and S8, see the ESI† for details). However, the use of light with wavelengths in the range of 395–850 nm as well as 305 and 260 nm, does not trigger photocyclization.

**Fig. 5:**
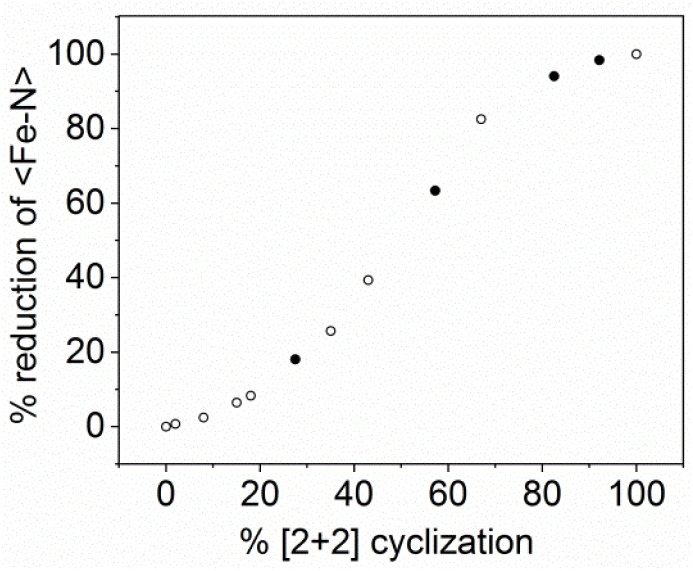
Relative 〈Fe–N〉 distance reduction (derived from 100(*d*(Fe–N)_%[2+2]_ − *d*(Fe–N)_LS_)/(*d*(Fe–N)_HS_ − *d*(Fe–N)_LS_)) *versus* the degree of [2 + 2] cyclization (estimated from occupancy factors of the cyclobutane derivative) determined at 100 K. First crystal – empty circles; second crystal – filled circles.

In the next experiment we performed the time-resolved X-ray measurements of the irradiated (365 nm) single crystal. Again, the irradiation was performed at 295 K, while X-ray diffraction was conducted at 100 K. The results show that the increase in the contribution of photo-cyclized molecules leads to an increase in the average 〈Fe–N〉 distance at 100 K indicating a change in spin crossover properties (Fig. 5 and S9a†). The dependence between the shortening of 〈Fe–N〉 distance and the degree of photocyclization indicates a strong relationship, taking on a sigmoidal shape over the entire range of the bond length. For coumarin fragments meeting the topochemical conditions, cyclization triggered by light irradiation is realized quantitatively resulting in 2. The process takes less than 1300 seconds to be completed (Fig. S9b†). The photoconversion proceeds according to first-order reaction kinetics (Fig. S9c†). Photocyclization resulting in 2 occurs without changes in chemical composition related to the presence of noncoordinated acetonitrile molecules.

The formation of the dimer in the photochemical process leads to the formation of the cyclobutane ring in 2, with the proximal lactone fragment orientation (Fig. 3b). This changes the tilt of the tetrazole ring regarding the coumarin fragment. It should be added that mutual orientations of coordinated tetrazole rings in 1 and 2 are rather similar. Photocyclization does not cause structural differentiation of the Fe(ii) centers and in the crystal lattice of 2 there is still one symmetrically independent Fe(ii) ion.

Despite structural changes in the ligand molecules, the composition of the first coordination sphere in 2 is only subtly altered in relation to the high spin form of 1. The Fe–N distances are equal to 2.187(1), 2.192(1) and 2.208(1) Å. The differences between Fe–N bond lengths in 2 (0.021 Å) is slightly lower in relation to 1 (0.026 Å). The average Fe–N distance in 2 (2.196 Å) is very close to the one found in 1 (2.193 Å). The N–Fe–N angles in 2 range from 87.16(4) to 92.84(4)°, while those in 1 adopt values from 87.18(6) to 92.82(6)°. At 250 K *Σ* adopts a value of 15.3° for 1 which increases to 22.7° after photoconversion. The shape distortion measure parameter^33^ for the FeN_6_ octahedron adopts a value of 0.065 at 250 K for 2 in relation to 0.046 for the starting spin crossover active compound 1. These slight differences within the geometry of the coordination octahedron may contribute to a greater stabilization of the HS form in 2 compared to 1.

The molecular structure of the free cyclobutane derivative reveals a distance of about 6.3 Å between both methylene carbon atoms C10⋯C21 (Fig. 3c). This distance decreases to 4.6 Å for 2, implying a significant strain in the 1D chain of the complex. The DFT estimated value of the corresponding stress upon coordination is 34 ± 4 kJ mol^−1^ (see the ESI† for details). The X-ray data reveal that upon coordination the reduction of the distances between the equivalent atoms of the lactone ring occurs (Fig. 3c). Another parameter displaying the strain upon the formation of a cyclobutane ring is the difference in torsion angles between the free dimerized ligand and its coordinated form in 2 (Table S4 and Fig. S10†).

Between two neighbouring complex cations in 1 there are two pairs of properly aligned coumarin moieties. Hence, photocyclization results in the formation of a double ligand bridge between two neighboring Fe(ii) ions. Propagation of this linking mode along the *a* direction leads to the formation of a one dimensional polymeric macrocation (Fig. 3b). After completion of [2 + 2] cyclization the *a* and *b* lattice parameters in 2 (80 K) adopt larger values in comparison to the ones in the LS structure of 1, whereas *c* adopts a lower value (Fig. S11†). The cumulative effect of photocyclization of 1 → 2 is reflected in very small changes in unit cell volumes from 1868.56(6) Å^3^ (80 K) to 1869.44(6) Å^3^ (80 K). Usually, the transition from the HS to LS phase is associated with a reduction in the volume of approximately 25 Å^3^ per complex molecule, including a decrease in the volume of the coordination octahedron at about 3 Å^3^ (∼25%).^34^ This means that the expected increase in the volume of 2 in relation to 1, caused by the appearance of HS Fe(ii) ions (100 K), was overbalanced by a reduction in the molecular volume of the organic component. The structural changes are associated with rebuilding of the network of intermolecular interactions (Table S3†). It is worth noting that intermolecular contacts in 1 established between coumarin fragments, not participating in the photoconversion, are still present in 2 (Fig. S7b†), facilitating transmission of perturbations produced by photoconversion to neighboring columns. Thus, besides stress produced by the compressed ligand molecule crystal lattice based effects can also contribute to the stabilization of the HS form.

An observed tendency for the stabilization of the HS form in 2 confirms the results of magnetic studies. Irradiation (365 nm at 295 K) of 1 induces photocyclization, resulting in the sample remaining in the high spin form down to 10 K (Fig. 2). Reduction of *χ*_M_*T* below 20 K can be attributed to zero field splitting (ZFS) in Fe(ii) ions. Thus, [2 + 2] photocyclization exerts an extremely strong effect resulting in switching from a compound capable of changing the spin state (1) to a product (2) with a stable high-spin state over the entire temperature range.

We have characterized two compounds, the mononuclear 1 and the 1D product of its photopolymerization 2, using methods such as X-rays, magnetic susceptibility and Mössbauer spectroscopy (Fig. S12, see ESI† for details). The spin crossover character of 1 and the HS state in the entire temperature range investigated for 2 were found. Furthermore, the X-ray and IR results for the illuminated crystals pointed to the coexistence of the mononuclear and polynuclear 1D species of 1 and 2 in one phase. Their ratio depends on the time of illumination, so it is of interest to investigate how the SCO behavior of both species is contingent on their ratio. While the X-ray diffraction experiments revealed only one independent Fe center in the mixed crystals obtained by illumination, Mössbauer spectroscopy allowed the observation of the Fe centres of both 1 and 2. Irradiation (365 nm) of 1 was carried out on a single layer of small crystals of similar size (*ca.* 0.3–0.5 mm), placed in a flat polyethylene capsule and then mounted on the cold finger of a cryostat. Upon an increase in temperature (starting from 50 K) the first changes caused by irradiation were noticed at 200 K; however photoconversion takes place to a greater extent at 225 K (8 h of irradiation) or at higher temperature. It should be noted that the observed increase in the contribution of the cyclic form during exposure at higher temperatures is a general characteristic of this process;^35^ however it must be taken into consideration that other factors can affect photodimerization.^36^ It leads to the appearance of a second high spin component (HS2) besides the starting form (HS1).

**Fig. 6:**
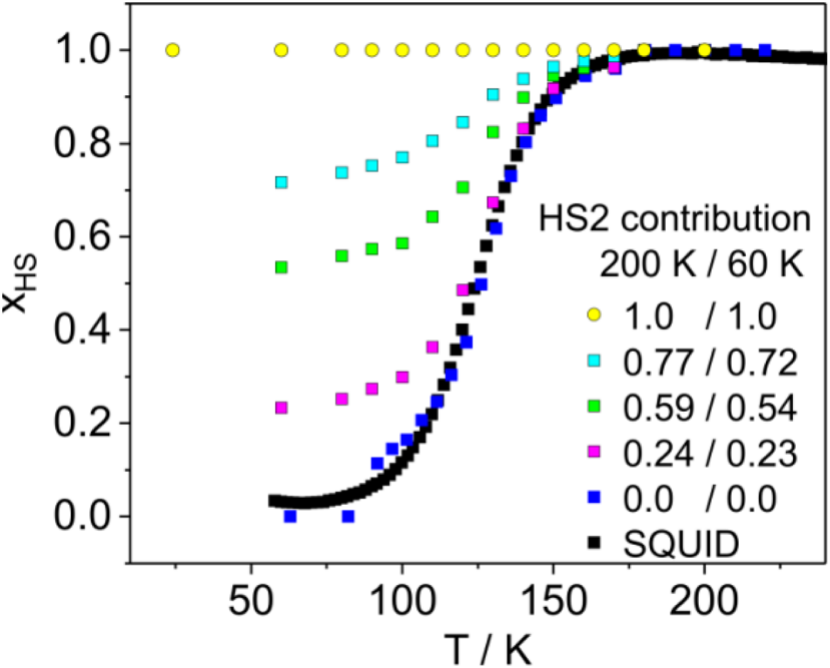
HS fraction *x*_HS_(*T*) and spin crossover dependences derived from Mössbauer spectra for a sample of compound 1 irradiated (*λ* = 365 nm, light power 10 mW, distance 80 mm) at different temperatures. *x*_HS_ has been taken as a total contribution of both high spin forms HS1 and HS2 derived from their relative spectral areas (*A*_HS1_ + *A*_HS2_)/(*A*_HS1_ +*A*_HS2_ + *A*_LS_). The inset shows contributions of the HS2 component derived from *A*_HS2_/(*A*_HS1_ + *A*_HS2_ + *A*_LS_) at 200 and 60 K. The HS fraction *vs.* temperature dependence for 1 derived from magnetic studies (1 K min^−1^, applied magnetic field: 1 T) is marked by black squares.

The HS2 component has a very similar value of the isomer shift *δ* = 1.14 mm s^−1^ and slightly greater value of the quadrupole splitting Δ*E*_Q_ = 2.86 mm s^−1^ (200 K, 54% conversion) compared to HS1 (Fig. S13†). It allowed us to independently monitor the modulation of spin crossover properties for both HS components (HS1 and HS2). In the first experiment about 23% of the initial HS1 form (estimated according to the *A*_HS2_/(*A*_HS2_ + *A*_HS1_) relationship at 200 K; with A being the spectral areas of HS1, and HS2, respectively) was converted by light into the HS2 form. Subsequently, variable temperature measurements in cooling mode from 200 to 60 K were carried out. It was established that the formation of the low spin form LS1 is correlated with the vanishing of the initial HS1 form, that is, the HS1 → LS1 process occurs. In the next experiments the degree of photoconversion was successively increased to 54%, 77% and 100% *via* irradiation at 250 K (16 h), 275 K (16 h) and 295 K (16 h), respectively. For 54% conversion the Mössbauer spectrum at 60 K is composed of the LS1 component (*δ* = 0.57 mm s^−1^ and Δ*E*_Q_ = 0.22 mm s^−1^) and HS2 component (*δ* = 1.20 mm s^−1^ and Δ*E*_Q_ = 3.17 mm s^−1^, Fig. S13†). The formation of the LS1 form was consistently associated with the disappearance of the HS1 form. Concomitantly the contribution of the HS2 form at lower temperatures was very similar to those found above the spin crossover temperature region (Fig. 6 inset). The results above point out that the formation of chains of 2 embedded into 1 does not significantly influence the SCO properties. 2 stays in the HS state throughout the entire temperature range, while *T*_1/2_ remains similar to 1. The last irradiation resulted in practically complete conversion to the HS2 form (Fig. S12 and S13†).

According to the single crystal X-ray diffraction studies the appearance of the first amounts of cyclized form of ligands is associated with the start of an increase in the Fe–N distance (Fig. 5). Assuming values typical for the high-spin form for complete [2 + 2] cyclization, we conclude that the appearance of the second high-spin component HS2 can be attributed to Fe(ii) ions linked with photocyclized ligand molecules. ^1^H NMR measurement of sample 2 (see the ESI† for details) showed that the molar ratio of the product (dimer) to the original ligand form is 1.98 : 1.

**Fig. 7:**
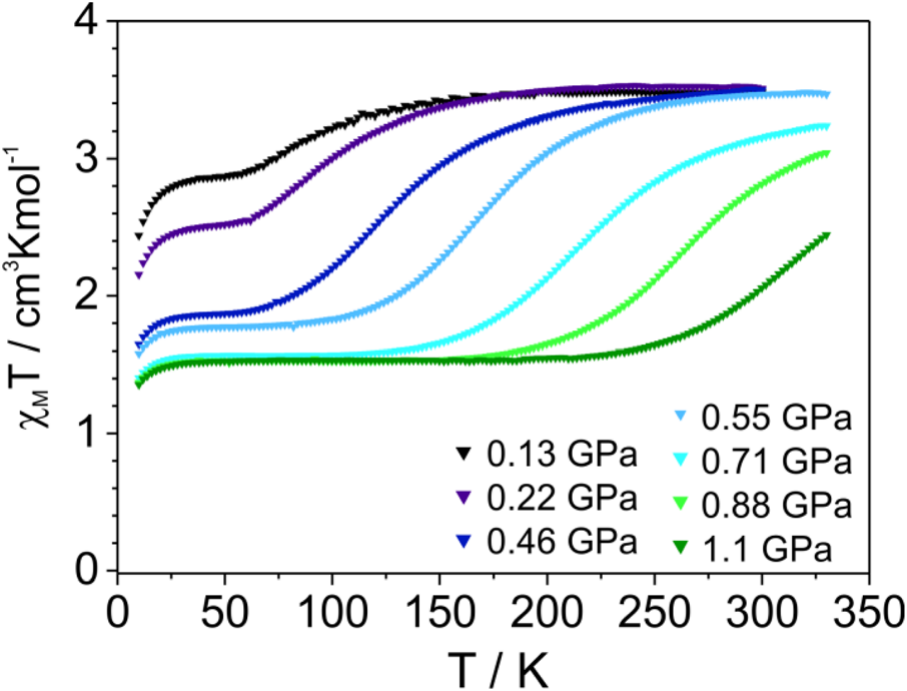
*χ*_M_ *T*(*T*) dependences (applied magnetic field: 1 T) for 2 recorded in cooling (1 K min^−1^) mode for indicated pressures.

It is worth noting that the estimated *T*_1/2_ values (50% of spin transition derived from comparison of areas of LS1 and HS1 forms according to the *A*_HS1_/(*A*_HS1_ + *A*_LS_) relation) practically do not depend on the degree of photoconversion (Fig. S14†). Thus, the course of spin crossover HS1 → LS1 remains practically unaffected. Hence, a question arises concerning the further consequences of local deformation, which acts directly on the [FeN_6_] chromophore, and concomitantly spreads to the entire crystal lattice, resulting in image pressure.^37^ Therefore, it may consequently affect the rest of the metal centers. A direct comparison of thermally induced spin crossover properties for HS1 and HS2 is not possible because the product of photoconversion HS2 remains in the stable high spin form during cooling. This means that the entropic part *T*Δ*S*_HL_ outweighs the Δ*H*_HL_ contribution corresponding to Δ*E*_HL_ = *E*_HS_ − *E*_LS_, where *E* denotes the sum of the electronic and vibronic energies of the spin states in the whole temperature range. Elevation of energy of the HS form *E*_HS_ could restore the accessibility towards the thermally induced spin crossover. This can be accomplished by applying pressure, which destabilizes the high spin form (*E*_HS_) to a larger degree as compared to the low spin form (*E*_LS_).^38^ To check the possibility of realizing HS2 → LS2 switching, the photoconverted sample 2 (obtained by 365 nm irradiation under monitoring with Mössbauer spectroscopy) was used for subsequent experiments. Indeed, magnetic susceptibility measurements performed under high pressure for 2 revealed an occurrence of thermally induced spin crossover (Fig. 7).

### Pressure and light induced spin crossover in 2

Initially, applying pressure causes an increase in the fraction of the low spin form, which exhibits spin crossover upon heating. The increase in the contribution of the low-spin form practically stops for pressures above 0.7 GPa and then gradual spin crossover is increasingly shifted to higher temperatures. At pressures of 0.7–1.1 GPa, about 50% of the molecules remain in the low spin form at lower temperatures, while a substantial amount of the low spin fraction still exists above 300 K. The presence of about half of the molecules in the high spin form can result from effective intrachain transmission of perturbations to neighboring spin crossover centers, stabilizing their high spin form.^39^ A similar behavior was found for the 1D system [Fe(bpym)(NCS)_2_] (bpym = 2,2′-bipyrimidine) where applying a pressure of 1.2 GPa leads to 50% of low spin species.^40^

**Fig. 8:**
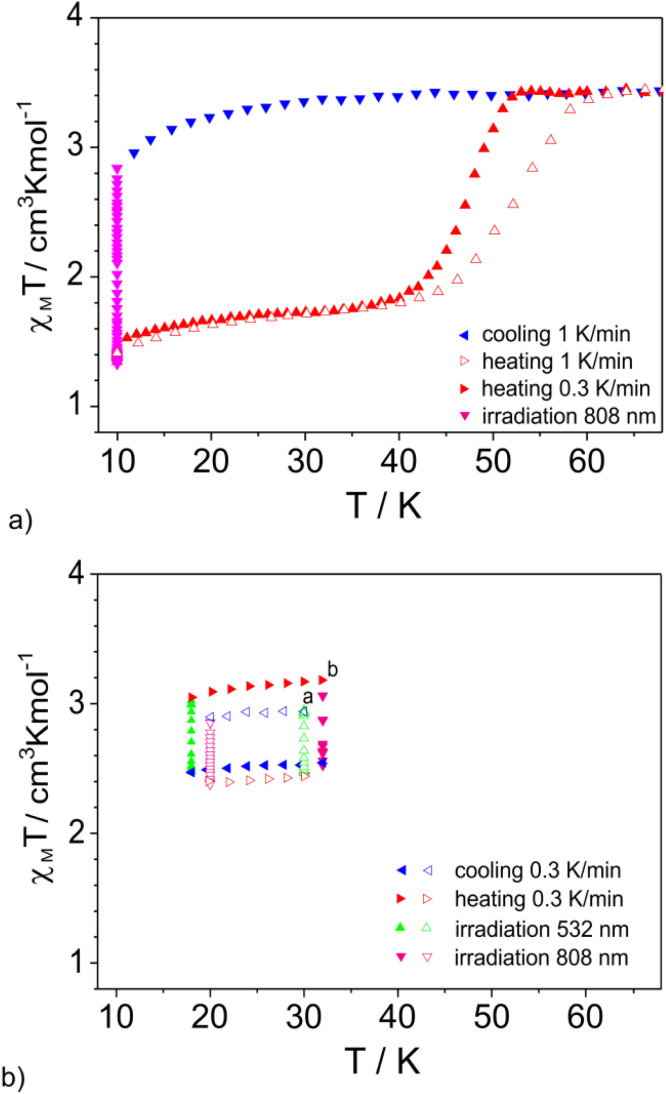
*χ*_M_ *T*(*T*) dependences recorded at 1 K min^−1^ (empty triangles) and 0.3 K min^−1^ (filled triangles) for 2 indicating the presence of “hidden hysteresis” (a). Clockwise (filled triangles) and anticlockwise (empty triangles) movement triggered by the combination of light irradiation and changes in the temperature (0.3 K min^−1^) is presented in (b). The vertex of the triangle (right, left, up, and down) indicates the direction of change. The starting point “a” was achieved after cooling 2 to 30 K and irradiating with light (808 nm). After completing the loop (empty triangles) at corner “a”, the temperature was elevated to 32 K and then followed by light irradiation (532 nm) to reach corner “b”. The applied magnetic field was equal to 1 T. To facilitate comparison of the position of *χ*_M_*T* dependences in (b) with respect to the hidden hysteresis (a), the same ranges of values of *χ*_M_*T* are used in figures (a) and (b).

The coexistence of spin crossover active species in both high spin and low spin forms over a wide temperature range indicates a small Δ*E*_HL_ difference. Thus, it can be concluded that 2 remains close to the spin crossover point. However, the transition is shifted below 50 K, where the thermally induced HS → LS process in Fe(ii) coordination compounds is kinetically inhibited. This also implies that, according to the inverse energy gap rule, it should be possible to populate the low spin state at the lowest temperatures by light irradiation (r-LIESST).^41^ Irradiation of sample 2 at 10 K (SQUID) with red light (808 nm) resulted in photoswitching (Fig. 8a) of about half of HS2 to the low spin form (denoted as LS2).

After saturation of magnetization the temperature was increased at a rate of 1 K min^−1^. Initially, the increase in temperature to 25 K resulted in an insignificant increase in *χ*_M_*T*, then followed by abrupt spin crossover above 40 K with *T*_1/2_ = 47 K (in this case *T*_1/2_ represents the temperature at which half of the Fe(ii) ions capable of thermally induced spin crossover undergo a change in spin state). Further heating did not induce any changes. The measurement performed in the heating mode with a lower temperature change rate (0.3 K min^−1^) causes the spin crossover to start at practically the same temperature, approximately 40 K, but it is more abrupt. Spatiotemporal studies performed for the LS2 phase revealed that at 20 K there are no observed changes in the *χ*_M_*T* value. At 30 K the value of *χ*_M_*T* changes less than 0.1 cm^3^ K mol^−1^ in 12 hours (Fig. S15†). At 40 K there occurs a transition from the LS2 to HS2 form; however it requires above 10 h. Thus, the observed value of *T*_1/2_ = 47 K (1 K min^−1^) is due to the slow kinetics of the spin crossover. Subsequent cooling did not trigger a thermally induced HS2 → LS2 transition down to 10 K. Re-exposing the sample to irradiation with light of wavelength 808 nm again resulted in the switching of about 50% of HS2 to the low spin form, indicating reproducibility of the bidirectional process (Fig. S16†). The contribution of the HS2 form for the sample remained unchanged after 12 h at 30 K. Thus, the combination of light-triggered HS2 → LS2 switching with the thermal transition LS2 → HS2 produces “hidden hysteresis”, a situation where the system exhibits bistability in a certain range of very low temperatures.^31^ It was established that a combination of temperature changes (0.3 K min^−1^) and changes in spin state, triggered by light irradiation, makes it possible to switch magnetic properties in any direction within the “hidden hysteresis” region while maintaining the magnetic stability of the macroscopic state reached (Fig. 8b).

Moreover, partial light induced HS2 → LS2 switching at 30 K, followed by very slow heating (0.1 K min^−1^) does not involve an increase in the contribution of the high spin or low spin form until reaching the thermally activated LS2 → HS2 transition region above 40 K. The value of *χ*_M_*T* for the sample partially switched at 30 K changes over 20 hours by less than 0.1 cm^3^ K mol^−1^ (Fig. S17†). According to Hauser's considerations,^38^ the generation of low spin molecules at very low temperature should favor the stabilization of the low spin state in 2 due to crystal lattice compression, leading to an increase in internal pressure. Thus, it should result in spontaneous conversion of the remaining HS molecules to the LS form. The results of high pressure studies of 2 revealed the occurrence of a very gradual spin crossover, indicating that at elevated pressure both spin forms appear next to each other, suggesting that both states have similar energies. This suggests that the expected tendency to stabilize the low-spin phase, as a result of lattice compression, may be counterbalanced by the effects originating from the presence of the strained form of the ligand. As a result, it is possible to achieve a steady state for any contribution of individual spin forms.

### DFT modelling of the molecules and the stress therein

To quantify the observed increase in stabilization of the high-spin state in the chain complex 2 obtained *via* photodimerization in relation to 1, DFT modelling was performed (see the ESI† for details, including the pdb of the optimised molecules and the movies showing the mode diagnostic for the dimerized lactone-based ligand). The calculated values of the electronic spin transition energy *E*_el_ (*E*_el_ = *E*_el_(HS) − *E*_el_(LS)) yields a value of 14 kJ mol^−1^ for the mononuclear cation and −4 kJ mol^−1^ per one cation in the pentanuclear model of photoswitched complex 2 (B3LYP/cep-31g with D3 dispersion correction, see the ESI†). This indeed reveals a significant stabilization of the HS state upon formation of the photoswitched polynuclear system. The analysis of the inner dimeric ligand strain in the obtained model structures of 2 in LS and HS states does not reveal significant and systematic differences, yielding an average increase in the bridging ligand stress of 2 ± 7 kJ mol^−1^ when going from the LS pentanuclear model to the HS one. Apparently the coordination to Fe(ii) leading to emergence of the 1D chain is unable to further increase the strain of the photodimerized ligand. Instead, it is the interligand strain that stabilizes HS states with longer Fe⋯Fe distances (see the ESI† for details). The calculation of the electronic energy of ligands in the geometry of pentanuclear LS and HS complexes, upon removal of the Fe cations and BF_4_^−^ anions shows that the ligand assembly corresponding to the HS structure is 110 kJ mol^−1^ lower in energy than that corresponding to the LS structure (see the ESI†). Additionally, the central Fe-atom in the LS model molecule suffers from the elongation of the Fe–N bonds (see the ESI†). We performed additional modelling of an analogue of the pentanuclear model of 2 in which the methylene linkage between tetrazole and coumarin phenyl was replaced with ethylene fragments for the dimerized ligands, 2b (see the ESI†) For this model *E*_el_ per one Fe atom decreases to −15 kJ mol^−1^ pointing towards further stabilization of the HS state. The corresponding change in the interligand strain leads to an energy difference of +122 kJ mol^−1^, again favouring the HS state. This indicates that the release of strain need not necessarily lead to a stabilisation of the LS state. Finally, a further entropic effect is to be noted. While the spin transition vibrational entropy Δ*S*_vib_ for 1 was calculated to be 72 J K^−1^ mol^−1^ at 298 K, the obtained value for 2 is 50 J K^−1^ mol^−1^ at 298 K, indicating a decrease in entropic stabilisation of the HS state upon photodimerization of the ligand.

## Conclusions

In this report, we present the preparation of the photoreactive coordination compound [Fe(L)_6_](BF_4_)_2_·4CH_3_CN (1) containing the coumarin-based ligand 7-(tetrazol-2-ylmethyl)coumarin (L). 1 exhibits thermally and light induced (*λ* = 532 nm) spin crossover. Its characteristic feature is the columnar arrangement of the complex cations [Fe(L)_6_]^2+^ and due to the flexibility of ligand molecules, the presence of pre-organized pairs of coumarin moieties allows photocyclization. Irradiation with light of *λ* = 365 nm triggers [2 + 2] photocyclization within the columns, resulting in the conversion from 0D (1) to 1D (2) structures. Structural transformation involves drastic changes in magnetic properties because the coordination polymer 2 remains in the high spin form (HS2) down to 10 K. This strong alteration of properties results from the formation of a stressed form of the cyclobutane derivative, which acts directly on the metal centers as well as on the crystal lattice. Applying high pressure restores the ability to trigger thermally induced (very gradual and incomplete) spin crossover in 2, however indicating that Δ*E*_HL_ still lies in the area of spin crossover. Spin state switching HS2 → LS2 can be accomplished with light of *λ* = 808 nm and the subsequent reverse process LS2 → HS2 with *λ* = 532 nm. The resulting low spin form is stable up to ∼40 K and a further increase in temperature triggers an abrupt thermally induced LS2 → HS2 transition indicating the occurrence of “hidden hysteresis”. Light induced spin state switching at *λ* = 532 or 808 nm allows us to achieve any spin state contribution (0.5 ≤ *γ*_HS_ ≤ 1), and a further change in temperature does not affect the high spin to low spin ratio within the “hidden hysteresis” region. Thus compound 2 exhibits extraordinary manageability of change in its magnetic properties to achieve a desired steady state.

To sum up, the presented approach, based on light induced structural modification demonstrates the possibility to produce a strained structural element capable of counterbalancing crystal lattice based effects resulting from a change in the spin state. This leads to a situation when a precisely planed macroscopic magnetic state can be reached and stabilized. A full understanding of the observed effect will require time dependent diffraction studies below 50 K for the systems with different degrees of light switching, revealing the kinetics of the possible relaxation processes.

## Data availability

Crystallographic data for compounds have been deposited at the CCDC under 2362570–2362571 (1), 2362572–2362574 (2), 2362575 (L) and 2362576 (L^[2+2]^). The experimental section, supporting data, computational analysis and movies have been uploaded as part of the ESI.†

## Author contributions

M. K.: compound synthesis, photoconversion experiments, FTIR microscopy measurements, data processing, analysis, data interpretation, and result presentation. M. W.: magnetic susceptibility data collection. M. S.: single crystal X-ray diffraction data collection. V. S. and J. A. W.: performed the DFT modelling. R. B.: supervision, financial support, manuscript preparation, Mössbauer spectroscopy data collection.

## Conflicts of interest

There are no conflicts to declare.

## Supplementary Material

SC-016-D4SC05587J-s001

SC-016-D4SC05587J-s002

SC-016-D4SC05587J-s003

SC-016-D4SC05587J-s004

SC-016-D4SC05587J-s005

SC-016-D4SC05587J-s006

SC-016-D4SC05587J-s007

SC-016-D4SC05587J-s008

SC-016-D4SC05587J-s009

SC-016-D4SC05587J-s010
