# Supplementary figures and images for: [2 + 2] Photocyclization converts thermally induced spin crossover effect into “hidden hysteresis” one
Source: Chem Sci. 2025 Mar 25;16(18):7884–93. doi: 10.1039/d4sc05587j (PMC11966535; doi:10.1039/d4sc05587j)

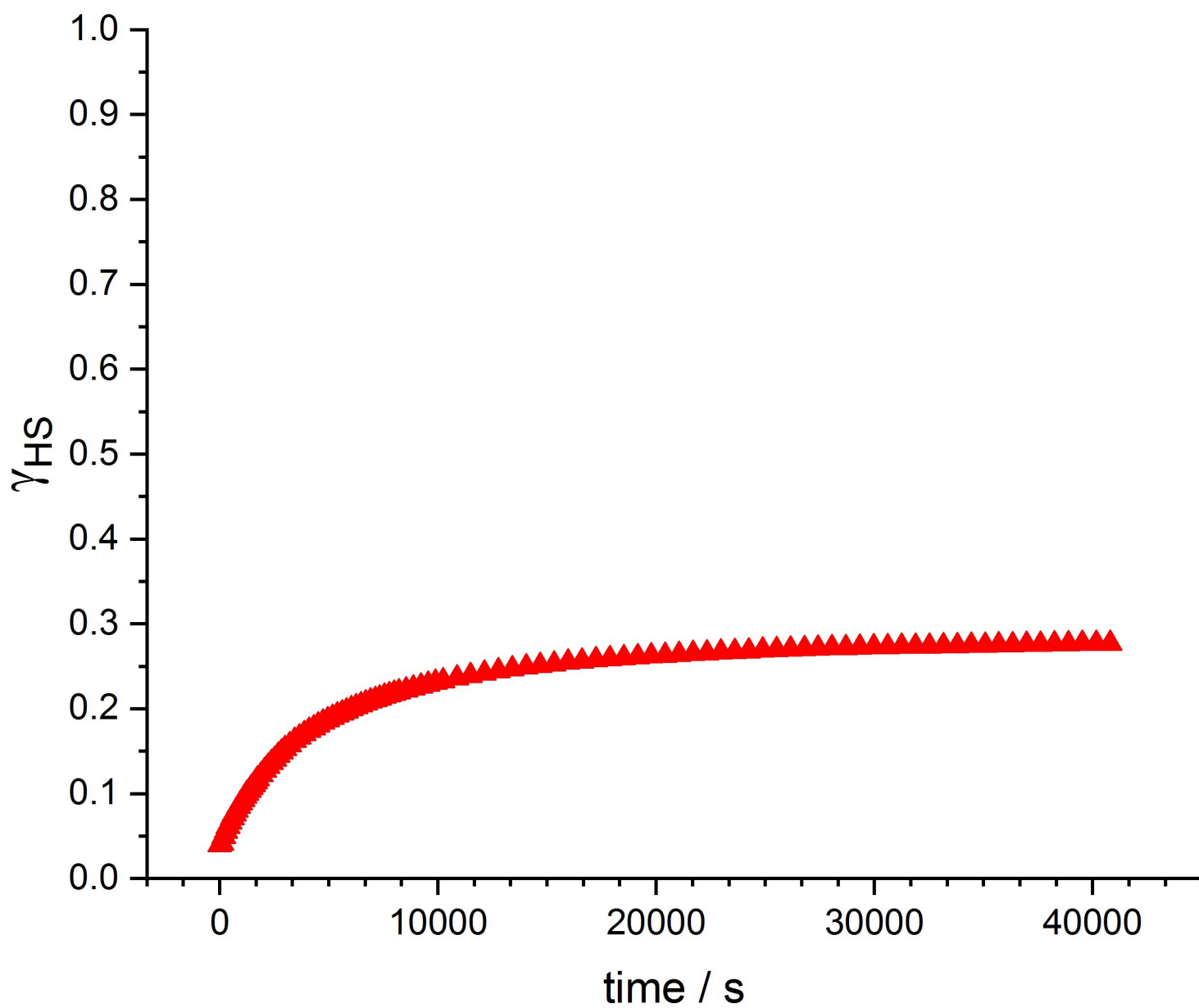

Supplement: SC-016-D4SC05587J-s004 [file SC-016-D4SC05587J-s004.pdf]
