# Supplementary material for: [2 + 2] Photocyclization converts thermally induced spin crossover effect into “hidden hysteresis” one
Source: Chem Sci. 2025 Mar 25;16(18):7884–93. doi: 10.1039/d4sc05587j (PMC11966535; doi:10.1039/d4sc05587j)
Supplement: SC-016-D4SC05587J-s010 [file SC-016-D4SC05587J-s010.pdf]

Supplementary Information (SI) for Chemical Science.
This journal is © The Royal Society of Chemistry 2025

[2+2] photocyclization converts thermally induced spin crossover into "hidden hysteresis" behavior

Marcin Kaźmierczak, [a] Marek Weselski, [a] Miłosz Siczek, [a] Juliusz A. Wolny, [b] Volker Schünemann [b] and Robert Bronisz [a] *

[a] *Faculty of Chemistry, University of Wrocław, F. Joliot-Curie 14, 50-383, Wrocław, Poland.*
[b] *Faculty of Physics, RPTU Kaiserslautern-Landau, Erwin Schrödinger Str. 46 67663 Kaiserlautern, Germany*

Contents: page

Materials and methods. 3
Scheme S1. Synthesis of 7-(tetrazol-2-ylmethyl)coumarin (**L**). 5
Synthesis of 7-methylcoumarin, 7-bromomethylcoumarin, 7-(tetrazol-2-ylmethyl)coumarin (**L**), $[Fe(L)_6](BF_4)_2\cdot 4CH_3CN$ (**1**), Photoconversion **1** $\rightarrow$ **2** , ^{1}H NMR characterization of ligands contribution in **2** and Isolation of $\mathbf{L^{[2+2]}}$. 5
Table S1. Crystallographic data for crystal structures of **1**, **2, L** and $\mathbf{L^{[2+2]}}$. 8
Table S2. Selected Fe-N, Fe…Fe distances, angles and torsional angles for **1** and **2**. 9
Table S3. Selected C-H···F, C-H···N and C-H···O interatomic distances [Å] and angles [°] for **1** (250 K, 80 K) and **2** (250 K, 80 K). 10
Table S4. Selected distances and torsional angles for [2+2] cyclization product derived. from crystal structure of free cyclobutane derivative ($\mathbf{L^{2+2}}$) and crystal structure of $\mathbf{L^2}$. 12
Figure S1. Thermogravimetric analysis of **1**. 13
Figure S2. Photo induced LS$\rightarrow$HS* switching in **1**. 13
HS*$\rightarrow$LS relaxation in 1. 14
Figure S3. Relaxation kinetics. $\gamma_{HS}(t)$ dependences at different temperatures for **1**. 14
Figure S4. Arrhenius plot of $\ln(k_{HL})$ versus 1/T for **1**. 15
Figure S5. α acceleration factor versus $1/T$ for HS*$\rightarrow$LS relaxation in **1**. 15
Figure S6. Crystal packing showing pillared arrangement in **1** 16
Figure S7. C-H···N and C-H···O intermolecular contacts in **1** and **2** 17
FTIR microscopy studies. 18
Figure S8. Spatio-temporal FTIR microscopy studies showing time evolution of spectra under irradiation (365 nm). 18
Figure S9. [2+2] photocyclization, average Fe-N distance and $\ln(c/c_0)$ versus time dependences derived from single crystal X-ray diffraction studies. 19
Figure S10. A comparison of selected distances and torsion angles of cyclobutane derivatives in crystal structures of free cyclobutane derivative and in the crystal structure of **2**. 20
Figure S11. Temperature dependence of lattice parameters and volume for **1** and **2**. Dependence of cell volume and lattice parameters vs degree of [2+2] photocyclization. 21
Mössbauer spectroscopy studies. 23
Figure 12. Mössbauer spectra of **1** (200 K and 60 K), **2** (60 K) and **1** recorded under light irradiation (532 nm) at 24 K. 24
Figure 13. Mössbauer spectra of **2** containing 23, 54, 77, and 100% of HS2 component. 25
Figure 14. HS1 $\rightarrow$ LS dependences derived from Mössbauer spectroscopy for different contributions of HS2 component. 26
Figure S15. Time evolution of $\chi_M T$ value in temperature 20, 30 and 40 K. 27
Figure S16. Bidirectional HS$\rightarrow$LS and LS$\rightarrow$HS switching in **2** 28
Figure S17. Temperature and time evolution of $\chi_M T$ value in temperature 30 K for two different partial HS2 $\rightarrow$ LS2 switching 29

DFT Modelling of the molecules and the stress therein 30
Figure S18. Optimized structures of the HS and LS dicationic models of **1** 30
Figure S19. Optimized structures of the HS and LS pentanuclear models of **2** 31
Figure S20. Optimized structure of the central unit of the LS pentanuclear models of **2** 32
Figure S21. Optimized structures of the HS and LS pentanuclear models of **2b** 33
Figure S22. Dimeric ligand assembly used for the calculation of the interligand repulsion in models of **2** 33
Figure S23. The molecular assembly used for the calculation of IR spectra of high-spin 1 involving the intermolecular interactions 34
Figure S24. Experimental and simulated spectra for high spin forms of **1** and **2**. 34
References 35

Materials and methods

Acetonitrile (HPLC grade, J.T. Baker) was dried by distillation over the calcium hydride. Sulfuric acid (98%, Chempur), malic acid (Aldrich), *m*-cresol (Merck), N-bromosuccinimide (Merck), potassium hydroxide (Chempur), sodium hydroxide (Eurochem BGD), sodium azide (Novichem) were used as delivered. Benzoyl peroxide (Merck) was recrystallized from anhydrous methanol (distilled over magnesium methoxide). Tetrazole was prepared according to previously described method.[1] Syntheses of iron(II) complexes were carried out under a nitrogen atmosphere using the standard Schlenk technique.

Elemental analyses for carbon, hydrogen and nitrogen were carried out using Vario ELcube analyzer. FTIR microscopy spectra in the range 4000-900 cm^{-1} (CaF_2 window) region were measured with a JASCO IRT-5200 infrared microscope. ^{1}H and ^{13}C NMR spectra were recorded on Bruker Avance III 500 MHz spectrometer. ESI-MS experiments were performed using Brucker Compact QTOF instrument (Bruker Daltonic, Germany) equipped with an ESI source. The instrument was operated in the positive-ion mode and calibrated with the ESI-L Low Concentration Tuning Mix (Agilent Technologies). The mass accuracy was better than 5 ppm. The acetonitrile/water/formic acid (50:50:0.1) mixture was used as solvents for recording the mass spectra. The obtained mass spectra were analyzed using the Bruker Data Analysis (Bruker Daltonic, Germany) software. Temperature dependent measurements of the magnetic susceptibility for **1** and **2** were carried out with a Quantum Design SQUID MPMS-XL-5 magnetometer under 1 T applied magnetic field. High pressures magnetic susceptibility measurements (1 T) were carried out with use of beryllium bronze high pressure cell (Quantum Design) Magnetometer Quantum Design MPMS3. Sample of **2** was placed in polyethylene capsule containing Pb wire and Daphne 7373 oil as pressure transmitting medium. The pressure inside the cell was measured (cooling mode at rate 0.05 K/min) in DC field (5 Oe) and calculated according to slope dT/dP of -0.379 $KGPa^{-1}$.[2] Light induced spin state switching experiments were carried out using laser lights of wave lengths 532 nm or 808 nm. Light power was adjusted to 5 and 3 mW, respectively, which allowed to maintain stable temperature. The weight of the samples were found by comparison with magnetic susceptibility of accurately weighed sample. Data have been corrected for the signal of the empty holder and for diamagnetism of the samples. Mössbauer spectra were recorded using ^{57}Co/Rh source (*ca.* 50 mCi) on POLON spectrometer working at constant acceleration mode. Lakeshore 200 device was used for temperature control. The sample of **1**, which was enriched with ^{57}Fe up to *ca.* 8%, was placed in the sealed polyethylene container of diameter 16 mm and mounted on a cold finger of helium cryostat (ARS DMX-20 cooling system). LIESST experiment (Mössbauer cryostat) was carried out using laser light (200 mW) of wave length 532 nm under continuous irradiation. Deconvolution of Mössbauer spectra was carried out by least-square fitting using Lorentzian functions (MOS software). The isomer shift δ and quadrupole splitting ΔE_Q were calculated in relation to α-Fe (295 K). TGA measurements were carried out with TGA-DSC Mettler-Toledo TGA/DSC 3. Photoinduced [2+2] cyclization was performed using 1 W light source (diode) of wave length 365 nm. Crystals of **1** intended for FTIR microscopy and SC-XRD studies were irradiated from a distance of 3 mm (1 W according to optical power meter LM 2, see main text for details). Macroscopic sample of **2** was obtained using Mössbauer spectroscopy monitoring. For this purpose a single layer of small crystals of similar size of **1** was placed in the sealed polyethylene container and placed in the cryostat. Irradiation (λ = 365 nm, light power 10 mW according to optical power meter LM 2) was carried out from a distance 80 mm until the HS1 component disappeared (see main text for details).

Single crystal X-ray data collection and structure determination.

The crystals of complex **1** suitable for single crystal X-ray diffraction studies were obtained directly from syntheses of macroscopic samples. Single crystals of **2** were obtained by irradiation (365 nm, 1W) of preselected single crystals of **1**. Single crystals of ligand L were obtained from crystallization from acetonitrile whereas crystals of its dimerized form **$L^{[2+2]}$** were grown from nitromethane. Crystals were covered with oil and placed on the loop. Structural studies were performed with a κ-geometry, four-circle XtaLAB Synergy-R DW (Rigaku) diffractometer with Mo-Kα (λ = 0.71073 Å) radiation and Hybrid Pixel Array Detector 150° (Rigaku). During low-temperature measurements, an Oxford Cryosystem cooling device was used. Low temperatures were achieved with a stream of cold nitrogen gas; the temperature stability was 0.1 K. CrysAlis^{Pro} software was used to control the measurement procedure, data reduction, determine and refine the lattice parameters.[3] The crystal structures **1**, **2**, **L** and **$L^{[2+2]}$** were solved using the SHELXS-2013 program and refined with the SHELXL-2019 program. Additionally OLEX2 was used as a refinement and analysis program.[4] Non-hydrogen atoms were refined with anisotropic thermal parameters and hydrogen atoms were introduced to the structure by appropriate rigid body constraints. CCDC 2362570-2362576 contain the supplementary crystallographic data for this paper. These data can be obtained free of charge from The Cambridge Crystallographic Data Centre via www.ccdc.cam.ac.uk/data_request/cif.

Computational methods.

The DFT calculations have been performed for the mononuclear model of **1** described in Discussion Chapter, based on the starting geometry taken from the X-ray data. In each case the structures of the high-spin and low-spin isomers have been optimized with the subsequent frequency calculation that did not reveal any imaginary frequencies. The modelling of the polynuclear chains of **2** were carried out using the pentanuclear model described as in the ESI Chapter "DFT Modelling of the molecules and the stress therein" below. The calculations were performed with Gaussian 16.[5] The B3LYP functional[6] with CEP-31g basis set[7] and Grimme dispersion correction[8] (keyword GD3 of Gaussian).

The movies depicting characteristic modes for ligands and complexes are placed as supporting materials. First part of the name denotes the model (monomeric or dimeric ligand, and the pentanuclear model of the HS 1D system), the number in the name denotes the calculated energy of the shown mode.

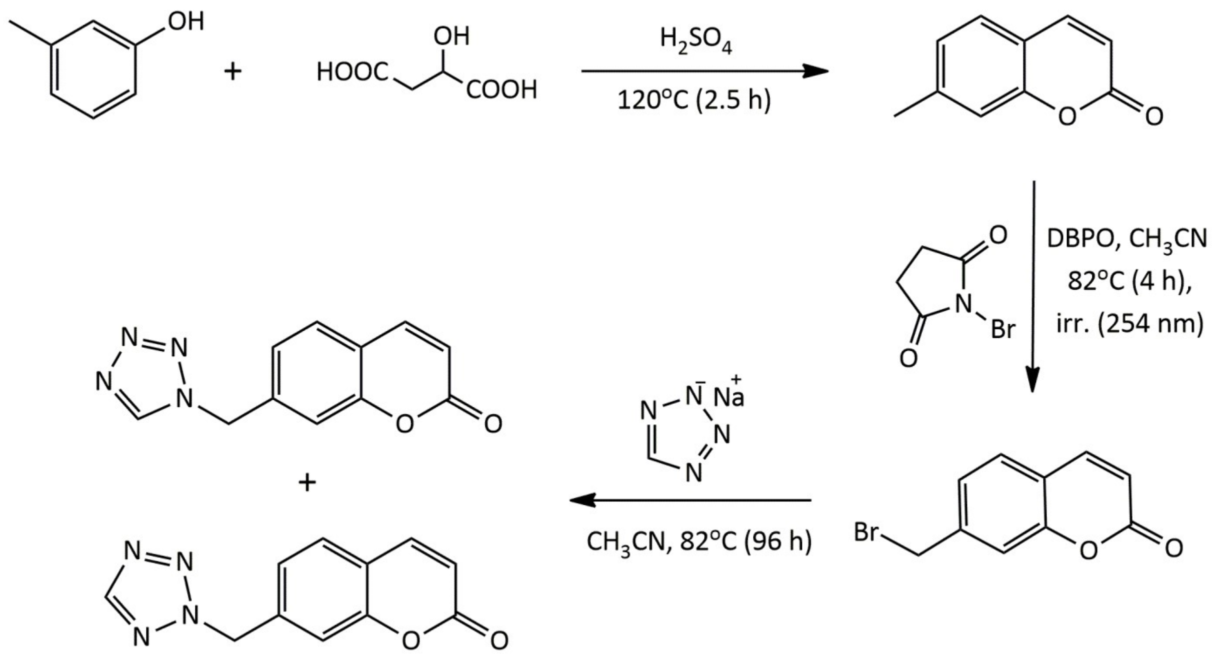

Scheme S1. Synthesis of 7-(tetrazol-2-ylmethyl)coumarin (**L**).

Synthesis of 7-methylcoumarin.

The synthesis was performed based on a literature-known procedure.[9]
To a solution of 2-hydroxysuccinic acid (25.5 g, 0.190 mol) in m-cresol (20.0 mL, 0.190 mol) at 0°C, concentrated sulfuric acid (50 mL) was added dropwise. The reaction mixture was stirred for 0.5 h at room temperature, then heated to 120°C and refluxed for 2.5 h. After cooling to room temperature, the mixture was poured into ice (100 g). The product was extracted with EtOAc (5 x 50 mL), washed with brine and dried over Na_2SO_4. After removing the solvent under vacuum, the residue was recrystallized with 95% ethanol (80 mL) to yield the final compound (11.9 g, 39.2%). Anal. found: C, 74.9; H, 5.2. Calc. for $C_{10}H_8O_2$ (M_w = 160.17): C, 75.0; H, 5.0. ^{1}H NMR (500 MHz, $CDCl_3$): δ 2.47 (s, 3H), 6.34 (d, 1H, J = 9.5 Hz), 7.08 (d, 1H, J = 8.0 Hz), 7.13 (s, 1H), 7.35 (d, 1H, J = 7.8 Hz), 7.66 (d, 1H, J = 7.8 Hz) ppm.

Synthesis of 7-bromomethylcoumarin.

The synthesis was performed based on a literature-known procedure.[10]
7-methylcoumarine (4.80 g, 30.0 mmol), N-bromosuccinimide (6.41 g, 36.0 mmol) and benzoyl peroxide (0.0375 g, 0.155 mmol) were added to a quartz round-bottom flask containing 80 mL of acetonitrile. The content was heated at reflux for 4 hours while being exposed to a mercury lamp. After this period of time, the reaction mixture was cooled to the room temperature and the solvent was removed under vacuum. The residue was treated with 1% aqueous potassium hydroxide (150 mL) and extracted with EtOAc (4 × 80 mL). The combined extracts were washed with brine, dried over Na_2SO_4 and then the solvent was removed in vacuo. The crude product was recrystallized from acetonitrile (18 mL) to give 2.37 g (Yield: 33%) of orange crystals. Anal. found: C, 50.2; H, 3.2. Calc. for $C_{10}H_7O_2Br$ (M_w = 239.07 g/mol): C, 50.2; H, 3.0. ^{1}H NMR (500 MHz, $CDCl_3$): δ = 4.52 (s, 2H), 6.43 (d, 1H, J = 9.5 Hz), 7.31 (m, 1H), 7.35 (s, 1H), 7.46 (d, 1H, J = 8.0), 7.69 (d, 1H, J = 9.5 Hz) ppm.

Synthesis of 7-(tetrazol-2-ylmethyl)coumarin (L).

A solution of NaOH (0.476 g, 11.9 mmol) in water (5 mL) was added to tetrazole (0.834 g, 11.9 mmol) in water (10 mL). The obtained clear solution was cooled, and water was removed to dryness under reduced pressure. The obtained sodium salt of tetrazole was suspended in acetonitrile (30 mL), and then 7-bromomethylcoumarine (2.37 g, 9.90 mmol) was added. The reaction mixture was stirred and refluxed for 7 days. Then, the solvent was evaporated under reduced pressure and the dried mixture was lixiviated with acetonitrile (8 × 20 mL). The extracts were combined and the solvent was removed in vacuo. The mixture of isomers was separated using column chromatography (10:0.035, CH_2Cl_2:CH_3CN) to obtain 0.810 g of a product (Yield: 35.9%). Anal. found: C, 57.8; H, 3.3; N, 24.8. Calc. for $C_{11}H_8N_4O_2$ (M_w = 228.21 g/mol): C, 57.9; H, 3.5; N, 24.6. ^{1}H NMR (500 MHz, CD_3CN): δ = 5.93 (s, 2H), 6.39 (d, 1H, J = 9.6 Hz), 7.25 (m, 1H), 7.30 (m, 1H), 7.60 (d, 1H, J = 8.0 Hz), 7.83 (d, 1H, J = 9.6 Hz), 8.63 (s, 1H). ^{13}C NMR (500 MHz, CD_3CN): δ = 56.7, 117.3, 118.1, 120.2, 125.2, 129.9, 138.8, 144.2, 154.4, 155.1, 161.0 ppm. MS m/z: 251.05 (calc. for $C_{11}H_8N_4O_2$ $[M+Na]^+$ 251.05).

Synthesis of $[Fe(L)_6](BF_4)_2\cdot 4CH_3CN$ (1).

$Fe(BF_4)_2\cdot 6H_2O$ (11.3 mg, 0.0334 mmol) was dissolved in 5 mL of dry, deoxygenated acetonitrile. Then solvent was removed in the stream of gaseous nitrogen. The process of pouring and removing of the same portions (4 mL) of dry acetonitrile was repeated four more times. The obtained residue was dissolved in acetonitrile (1 mL) and then added to a solution of **L** (45.6 mg, 0.2 mmol) in acetonitrile (2 mL). The resulted colorless solution was left under nitrogen atmosphere in a closed Schlenk flask in 4^0C. The colorless crystals grew within 7 days. Yield 20.4% (12.0 mg). Anal. found: C, 50.6; H, 3.1; N, 22.4%. Calc. for $C_{74}H_{60}B_2F_8FeN_{28}O_{12}$ (M_w = 1762.97 g/mol): C, 50.4; H, 3.4; N, 22.3%.

Photoconversion 1 $\rightarrow$ 2

In order to obtain a macroscopic sample of compound **2**, 30 mg of compound **1** was placed in a plastic container used for measuring Mössbauer spectra. The sample was irradiated (365 nm, 1 W diode, distance from sample 80 mm, measured light power 10 mW) in a Mössbauer cryostat, with temperature measurements of Mössbauer spectra performed after each exposure. A detailed description of the procedure can be found in the main text. Subsequent irradiations were continued until the component of the spectrum corresponding to the starting compound **1** disappeared.

Contribution of product of photoconversion (dimer) in relation to initial ligand form was besides single crystal X-ray diffraction studies, estimated also by ^{1}H NMR spectroscopy (see spectrum below). For this purpose 4.0 mg of sample **2** was dissolved at room temperature in 0.5 ml of the mixture deuterated acetonitrile/ deuterated water (10:1 – v/v). The molar ratio of dimer (signals marked as "*d*" on spectrum) to monomer (signals marked as "*m*") was estimated to be 1.98:1 (based on integration of signals of protons of methylene linkers), which means that cyclization of molecules showing the appropriate topology occurred practically quantitatively.

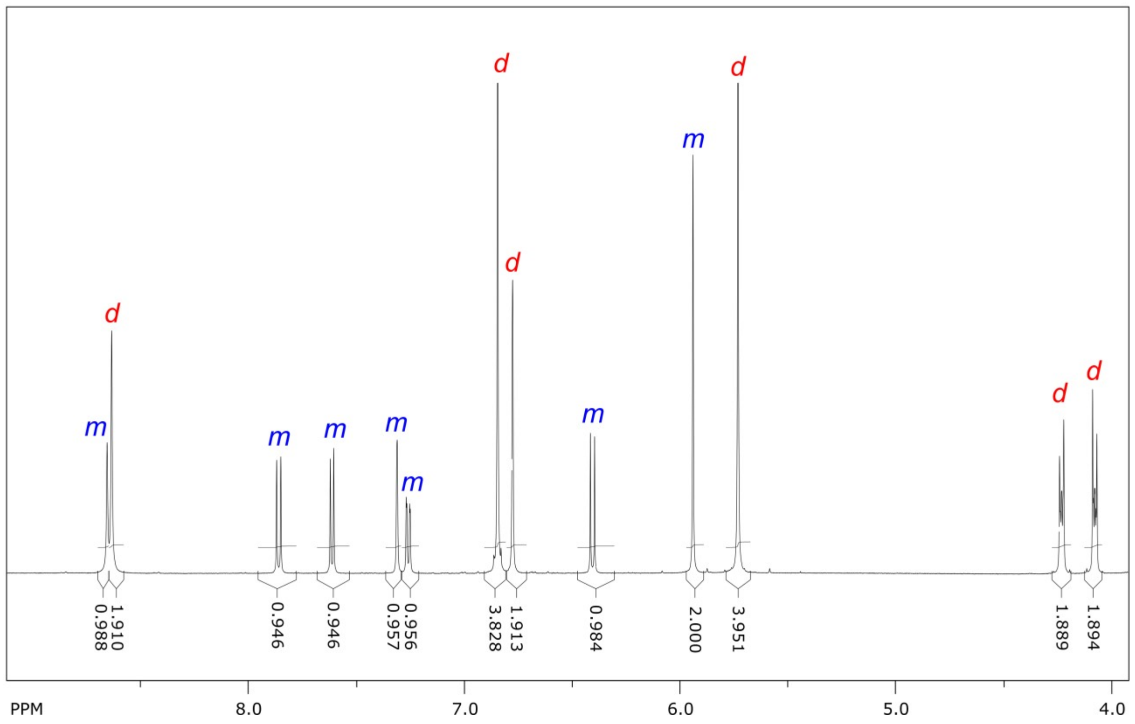

Isolation of L[2+2]

Dimerized ligand (**L[2+2]**) isolated from photoconverted complex (120 mg) was dissolved in 2 ml of boiled acetonitrile. To the resulted cooled, clear solution about 3 ml water was added. White precipitate was separated and recrystallized from minimal amount (*ca.* 1 ml) of hot mixture of water/acetonitrile (1/2 : v/v). After cooling white precipitate was washed with diethyl ether and dried on air. It was obtained 53 mg of white powder. ^{1}H NMR (500 MHz, CD_3CN): δ = 4.07 (m, 1H), 4.24 (m, 1H), 5.73 (s, 2H), 6.79 (s, 1H), 6.85 (s, 2H), 8.61 (s, 1H). ^{13}C NMR (500 MHz, CD_3CN): δ = 40.4, 40.6, 56.4, 117.6, 119.6, 125.0, 131.2, 136.4, 153.1, 154.3, 165.5 ppm. MS m/z: 479.12 (calc. for $C_{22}H_{16}N_8O_4$ $[M+Na]^+$ 479.12).

Table S1. Crystallographic data for crystal structures of **1, 2, L** and **L[2+2]** .

T/K	**1**<br>**250 K**	**1**<br>**80 K**	**2**<br>**250 K**	**2**<br>**80 K**	**L**<br>**100 K**	**L[2+2]**<br>**100 K**
CCDC number	2362570	2362571	2362572	2362574	2362575	2362576
Chemical formula	$C_{74}H_{60}B_2F_8$ $FeN_{28}O_{12}$	$C_{74}H_{60}B_2F_8$ $FeN_{28}O_{12}$	$C_{74}H_{60}B_2F_8$ $FeN_{28}O_{12}$	$C_{74}H_{60}B_2F_8$ $FeN_{28}O_{12}$	$C_{11}H_8N_4O_2$	$C_{22}H_{16}N_8O_4$
Formula Mass	1762.97	1762.97	1762.97	1762.97	228.21	456.43
Crystal system	triclinic	triclinic	triclinic	triclinic	triclinic	triclinic
Space group	*P* -1	*P* -1	*P* -1	*P* -1	*P* -1	*P* -1
Z	1	1	1	1	2	2
Unit cell dimensions<br>*a*/Å<br>*b*/Å<br>*c*/Å<br>*α*/°<br>*β*/°<br>*γ*/°	 11.012(6)<br>13.113(7)<br>14.893(8)<br>80.80(4)<br>82.41(4)<br>67.88(3)	 10.710(2)<br>12.918(3)<br>14.735(4)<br>81.53(3)<br>82.54(3)<br>68.41(2)	 10.816(3)<br>13.308(4)<br>14.753(4)<br>93.25(2)<br>99.70(2)<br>112.47(3)	 10.747(2)<br>13.208(3)<br>14.680(3)<br>93.24(2)<br>99.84(2)<br>113.19(3)	 4.283(4)<br>9.818(8)<br>12.062(9)<br>84.15(4)<br>81.63(4)<br>79.92(3)	 6.597(2)<br>12.473(4)<br>12.691(4)<br>101.07(3)<br>99.32(3)<br>97.21(2)
Unit cell volume/$Å^3$	1960.7(2)	1868.56(6)	1917.18(8)	1869.44(6)	492.5(7)	998.02(5)
μ (mm^{-1})	0.292	0.306	0.299	0.306	0.111	0.919
No. of measured reflections	31358	29594	38314	36399	3680	14010
No. of independent reflections	11164	10627	13027	12575	2253	3512
Theta range for data collection [º]	1.689<br>to<br>29.999	2.052<br>to<br>29.999	2.088<br>to<br>31.998	2.057<br>to<br>31.999	3.425<br>to<br>28.817	3.615<br>to<br>66.573
Index ranges	$-15 \le h \le 15$<br>$-17 \le k \le 18$<br>$-20 \le l \le 20$	$-15 \le h \le 15$<br>$-17 \le k \le 18$<br>$-20 \le l \le 20$	$-16 \le h \le 16$<br>$-19 \le k \le 19$<br>$-21 \le l \le 21$	$-15 \le h \le 16$<br>$-19 \le k \le 19$<br>$-21 \le l \le 21$	$-5 \le h \le 5$<br>$-13 \le k \le 12$<br>$-16 \le l \le 9$	$-7 \le h \le 7$<br>$-14 \le k \le 14$<br>$-15 \le l \le 15$
R_{int}	0.0267	0.0277	0.0248	0.0257	0.0301	0.0574
Final R_1 values ($I > 2\sigma(I)$)	0.0418	0.0367	0.0410	0.0371	0.0514	0.0514
Final $wR(F^2)$ values ($I > 2\sigma(I)$)	0.1016	0.0885	0.0989	0.0922	0.0931	0.1452
Final R_1 values (all data)^a	0.0555	0.0450	0.0571	0.0463	0.0930	0.0555
Final $wR(F^2)$ values (all data)^b	0.1075	0.0918	0.1053	0.0964	0.1083	0.1497
Goodness-of-fit on F^2	1.058	1.060	1.041	1.036	1.037	1.077
Largest diff. peak and hole [$eÅ^{-3}$]	0.397<br>and<br>-0.362	0.426<br>and<br>-0.533	0.304<br>and<br>-0.272	0.472<br>and<br>-0.304	0.291<br>and<br>-0.227	0.455<br>and<br>-0.370

Table S2. Selected Fe-N, Fe…Fe distances, angles and torsional angles for **1** and **2**.

**compound**	**1**		**2**	
**T [K]**<br><br>**spin state**	**250 K**<br><br>**HS**	**80 K**<br><br>**LS**	**250 K**<br><br>**HS**	**80 K**<br><br>**HS**
Σ	15.3	15.0	22.7	23.7
Fe1-N4<br>Fe1-N4[i]	2.208(2)	2.016(1)	 2.208(1)	 2.195(1)
Fe1-N8	2.187(2)	1.993(1)	2.192(1)	2.188(1)
Fe1-N12	2.183(2)	1.984(1)	2.187(1)	2.180(1)
N4-Fe1-N8<br>N4[i]-Fe1-N8	89.75(6)	89.04(5)	 89.10(5)	 89.08(4)
N4-Fe1-N8[ii]<br>N4[iii]-Fe1-N8	90.25(6)	90.96(5)	 90.90(5)	 90.92(4)
N4-Fe1-N12<br>N4[i]-Fe1-N12	89.26(6)	89.63(5)	 88.07(5)	 87.92(4)
N4-Fe1-N12[ii]<br>N4[iii]-Fe1-N12	90.74(6)	90.37(5)	 91.93(5)	 92.08(4)
N8-Fe1-N12	87.18(6)	87.58(5)	87.16(4)	87.08(4)
N8-Fe1-N12[ii]	92.82(6)	92.42(5)	92.84(4)	92.92(4)
N2-C10-C7	114.0(1)	114.40(1)	112.6(1)	112.6(1)
N6-C21-C18	110.9(1)	111.5(1)	112.8(1)	112.61(9)
N10-C32-C29	111.7(1)	111.8(1)	110.7(1)	110.67(9)
N7-N6-C21-C18	91.4(2)	87.8(1)	-97.2(1)	-97.7(1)
N5-N6-C21-C18	-87.9(2)	-94.7(1)	83.6(2)	82.9(1)
N3-N2-C10-C7	78.3(2)	80.2(1)	-85.6(2)	-83.8(1)
N1-N2-C10-C7	-104.4(2)	-104.9(1)	93.3(2)	93.8(1)
C6-C7-C10-N2	17.1(2)	16.4(2)	-0.5(2)	-1.0(2)
C8-C7-C10-N2	-165.2(1)	-165.8(1)	-179.0(1)	-179.5(1)
C17-C18-C21-N6	-75.7(2)	-74.0(1)	88.6(2)	88.6(1)
C19-C18-C21-N6	104.7(2)	106.5(1)	-94.6(1)	-94.2(1)
N11-N10-C32-C29	131.4(1)	136.2(1)	-126.3(1)	-124.6(1)
N9-N10-C32-C29	-53.0(2)	-49.3(2)	56.3(2)	57.9(1)
C28-C29-C32-N10	115.9(2)	117.8(1)	-106.4(2)	-105.5(1)
C30-C29-C32-N10	-65.2(2)	-63.0(1)	71.9(2)	72.3(1)
Fe1···Fe1[iv]	11.012(6)	10.710(2)	10.8160(3)	10.7470(2)
Fe1···Fe1[v]	13.113(7)	12.918(3)	13.3080(4)	13.2080(3)
Fe1···Fe1[vi]	14.893(8)	14.735(4)	14.7530(4)	14.6800(3)

[i] x-1, y, z; [ii] -x, -y+2, -z+1; [iii] -x+1, -y+2, -z+1; [iv] x+1, y, z; [v] x, y+1, z; [vi] x, y, z+1.

Table S3. Selected C-H···F (**a**), C-H···N, and C-H···O (**b**) interatomic distances [Å] and angles [°] for **1** (250 K, 80 K) and **2** (250 K, 80 K). Distances higher then 3.5 Å were included to enable comparison.

a)

**compound**<br>**T [K]**<br>**spin state**	**1**<br>**250 K**<br>**HS**		**1**<br>**80 K**<br>**LS**		**2**<br>**250**<br>**HS K**		**2**<br>**80 K**<br>**HS**	
	C...A [Å]	C-H...A [°]	C...A [Å]	C-H...A [°]	C...A [Å]	C-H...A [°]	C...A [Å]	C-H...A [°]
C2-H2···F1[i]	3.364(3)	151.7	3.302(2)	146.7				
C2-H2···F1[x]					3.280(2)	130.4	3.193(2)	127.9
C2-H2···F2[i]	3.221(6)	150.1	3.241(9)	153.8				
C2-H2···F2[x]					3.369(9)	173.9	3.410(2)	172.2
C6-H6···F4[ii]	3.546(9)	168.2	3.35(1)	164.5				
C6-H6···F4[vii]					3.70(1)	149.1	3.648(2)	147.3
C6-H6···F4A[ii]	3.501(7)	153.2	3.42(1)	157.2				
C6-H6···F4A[vii]					3.61(2)	145.0		
C11-H11···F1[v]	3.507(3)	141.8	3.354(2)	141.1				
C11-H11···F1					4.294(2)	139.0	4.284(2)	139.2
C11-H11···F3[v]	3.61(1)	121.7	3.601(7)	120.5				
C11-H11···F3					3.192(9)	135.9	3.203(2)	138.9
C17-H17···F1[vii]	3.548(3)	141.0	3.472(2)	142.5	3.520(2)	152.2	3.462(8)	153.1
C21-H21A···F4[vii]	3.384(6)	150.8	3.28(1)	147.9				
C21-H21A···F4[vii]					3.48(2)	151.2	3.444(2)	148.9
C21-H21A···F4A[vii]	3.53(1)	144.1	3.41(1)	145.4	3.650(2)	146.0		
C21-H21A···F3A[vii]	3.79(1)	168.4	3.83(1)	166.8	3.48(1)	165.3		
C32-H32A···F1[v]	4.173(3)	156.6	3.940(2)	153.7	3.469(2)	172.5	3.431(2)	171.7
C33-H33···F2	3.277(8)	132.7	3.262(8)	125.4	3.122(8)	143.6	3.145(1)	144.6
C33-H33···F2A	3.251(7)	134.4	3.217(9)	125.6	3.30(2)	144.6		
C33-H33···F4	3.330(7)	167.3	3.26(1)	164.5	3.41(2)	160.8	3.378(2)	157.5
C33-H33···F4A	3.488(8)	156.6	3.35(1)	157.8	3.370(2)	156.9		
C34-H34C···F2	3.153(7)	137.4	3.093(8)	143.7				
C34-H34C···F2[iv]					3.492(1)	143.0	3.453(2)	142.9
C34-H34C···F2A	3.237(8)	146.3	3.131(9)	148.1				
C36-H36A···F1	3.325(3)	106.6	3.269(2)	103.1	3.331(3)	126.7	3.262(2)	125.9
C36-H36C···F2	3.49(1)	119.8	3.286(9)	112.3	3.311(1)	111.1	3.211(2)	115.6
C36-H36C···F2A	3.341(7)	109.8	3.252(8)	107.7	3.17(2)	114.5		

[i] x-1, y, z+1; [ii] -x, -y+2, -z+1; [iii] -x, -y+1, -z+2; [iv] -x, -y+1, -z+1; [v] x-1, y, z; [vi] x, y, z+1; [vii] -x+1, -y+2, -z+1; [viii] -x+1, -y+1, -z+1; [ix] x+1, y, z; [x] x, y, z-1; [xi] -x, -y+2, -z; [xii] x+1, y, z-1 [xiii] -x+2, -y+1, -z+1; [xiv] -x+1, -y+1, -z; [xv] -x, -y+1, -z; [xvi] -x, -y+2, -z+2.

b)

compound<br>T [K]<br>spin state	**1**<br>**250 K**<br>**HS**		**1**<br>**80 K**<br>**LS**		**2**<br>**250**<br>**HS K**		**2**<br>**80 K**<br>**HS**	
	C...A [Å]	C-H...A [°]	C...A [Å]	C-H...A [°]	C...A [Å]	C-H...A [°]	C...A [Å]	C-H...A [°]
C10-H10A···O2^{iii}	3.356(3)	144.1	3.342(2)	142.5				
C10-H10A···O2^{xiv}					3.433(2)	136.6	3.416(2)	139.4
C11-H11···N7^{ii}	3.152(3)	126.3	2.985(2)	125.6				
C11-H11···N7^{vii}					3.239(2)	125.9	3.210(2)	125.9
C13-H13···N9^{vi}	3.256(3)	139.6	3.196(2)	138.0				
C13-H13···N9^{x}					3.488(2)	156.7	3.428(2)	157.0
C16-H16···O5^{ii}	3.675(3)	157.5	3.599(2)	156.7	3.412(2)	138.2	3.363(2)	137.2
C21-H21B···N1^{ix}	3.347(3)	125.1	3.322(2)	120.3				
C21-H21B···N1					3.228(2)	126.1	3.176(2)	127.2
C22-H22···N14^{viii}	3.370(3)	175.2	3.291(2)	164.3				
C22-H22···N14^{iv}					3.356(3)	177.6	3.35(2)	177.1
C24-H24···N3^{x}	3.434(3)	124.2	3.360(2)	123.5				
C24-H24···N3^{i}					3.321(2)	117.7	3.270(2)	114.8
C25-H25···O4^{viii}	3.516(3)	144.7	3.370(2)	146.0				
C25-H25···O4^{iv}					3.508(2)	143.5	3.463(2)	143.5
C27-H27···O3^{viii}	3.577(3)	154.0	3.498(2)	154.6				
C27-H27···O3^{iv}					3.547(2)	156.0	3.500(2)	155.5
C27-H27···O4^{viii}	3.488(3)	147.7	3.462(2)	143.3				
C27-H27···O4^{iv}					3.368(2)	152.0	3.339(2)	151.0
C28-H28···N13	3.463(3)	145.5	3.420(2)	146.5				
C28-H28···N13^{iv}					3.397(2)	150.7	3.351(2)	149.7
C30-H30···O6^{xi}	3.314(2)	116.2	3.250(2)	118.7				
C30-H30···O6^{xvi}					3.243(2)	94.3	3.207(2)	92.5
C34-H34A···O4^{x}	3.434(4)	141.6	3.377(2)	139.3				
C34-H34A···O4^{xv}					3.636(3)	131.3	3.585(2)	130.0
C34-H34B···N5^{viii}	3.329(3)	116.4	3.312(2)	117.8				
C34-H34B···N5					3.236(3)	104.2	3.161(2)	103.1
C36-H36A···O2^{xii}	3.624(3)	141.1	3.545(2)	143.3				
C36-H36A···O2^{vi}					3.481(3)	140.6	3.393(2)	139.5

^{i} x-1, y, z+1; ^{ii} -x, -y+2, -z+1; ^{iii} -x, -y+1, -z+2; ^{iv} -x, -y+1, -z+1; ^{v} x-1, y, z; ^{vi} x, y, z+1; ^{vii} -x+1, -y+2, -z+1; ^{viii} -x+1, -y+1, -z+1; ^{ix} x+1, y, z; ^{x} x, y, z-1; ^{xi} -x, -y+2, -z; ^{xii} x+1, y, z-1 ^{xiii} -x+2, -y+1, -z+1; ^{xiv} -x+1, -y+1, -z; ^{xv} -x, -y+1, -z; ^{xvi} -x, -y+2, -z+2.

Table S4. Selected distances and torsional angles for [2+2] cyclization product derived from crystal structure of free cyclobutane derivative (**L^{2+2}**) and crystal structure of **L^2**.

	L^{2+2}<br><br>**100 K**	L^2<br><br>**100 K**
N1-N5	6.091(3)	4.934(1)
N2-N6	5.718(3)	5.431(1)
N3-N7	4.988(3)	7.095(1)
N4-N8	4.741(3)	7.695(1)
C11-C22	5.560(3)	6.497(2)
C10-C21	6.281(3)	4.601(2)
C7-C18	5.157(3)	4.113(2)
C6-C17	4.434(3)	3.798(2)
C8-C19	4.924(3)	4.011(2)
C9-C20	3.845 (3)	3.412(2)
C5-C16	3.360(2)	3.191(2)
C4-C15	2.943(2)	2.841(2)
C3-C14	1.591(2)	1.600(2)
O1-O3	3.870(2)	3.466(1)
C2-C13	1.571(2)	1.574(2)
C1-C12	2.927(3)	2.852(2)
O2-O4	3.343(2)	3.341(1)
N1-N2-C10-C7<br>N5-N6-C21-C18	-68.7(2)<br>-83.8(2)	93.8(1)<br>82.8(1)
N2-C10-C7-C6<br>N6-C21-C18-C17	74.2(2)<br>153.3(2)	-1.2(2)<br>88.7(1)
N2-C10-C7-C8<br>N6-C21-C18-C19	-107.1(2)<br>-29.0(2)	-179.4(1)<br>-94.1(1)
C10-C7-C6-C5<br>C21-C18-C17-C16	179.6(1)<br>175.0(2)	-173.7(1)<br>172.7(1)
C10-C7-C8-C9<br>C21-C18-C19-C20	-179.6(1)<br>-174.8(2)	174.0(1)<br>-172.8(9)
C7-C6-C5-C4<br>C18-C17-C16-C15	-0.1(2)<br>-0.6(3)	0.6(2)<br>-1.0(2)
C7-C8-C9-C4<br>C18-C19-C20-C15	0.1(2)<br>0.3(3)	-1.0(2)<br>1.2(2)
C6-C5-C4-C3<br>C17-C16-C15-C14	177.6(1)<br>-171.1(2)	165.4(1)<br>-163.3(1)
C5-C4-C3-C14<br>C16-C15-C14-C3	-70.7(2)<br>89.5(2)	-72.6(1)<br>89.7(1)
C8-C9-O1-C1<br>C19-C20-O3-C12	175.4(1)<br>-179.7(1)	172.3(1)<br>-179.1(1)
C4-C3-C14-C15	23.8(2)	17.5(1)
C9-O1-C1-C2<br>C20-O3-C12-C13	-7.4(2)<br>-2.5(2)	-14.2(2)<br>7.2(2)
C9-O1-C1-O2<br>C20-O3-C12-O4	174.0(1)<br>178.1(1)	168.0(1)<br>-176.0(1)
C1-C2-C13-C12	22.8(2)	16.1(1)
O1-C1-C2-C13<br>O3-C12-C13-C2	-82.4(2)<br>115.4(2)	-76.6(1)<br>104.5(1)

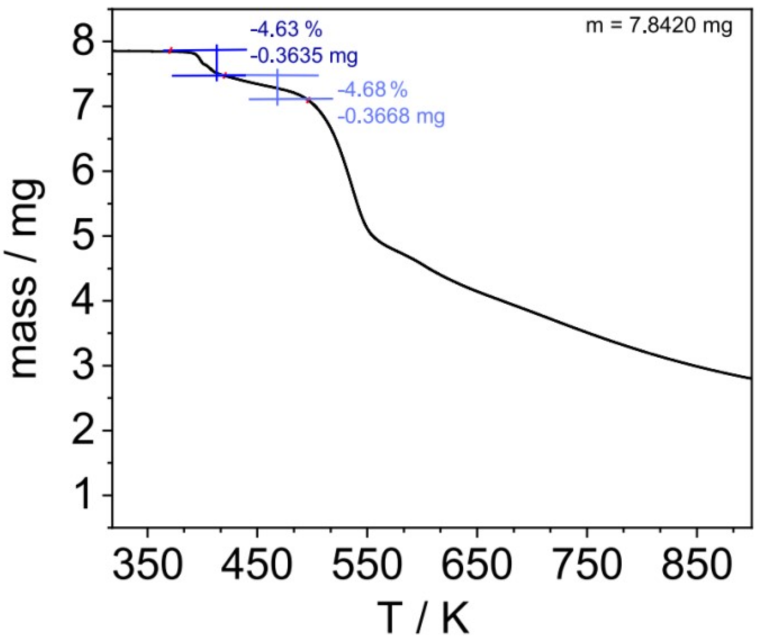

Figure S1. Thermogravimetric analysis of **1**.

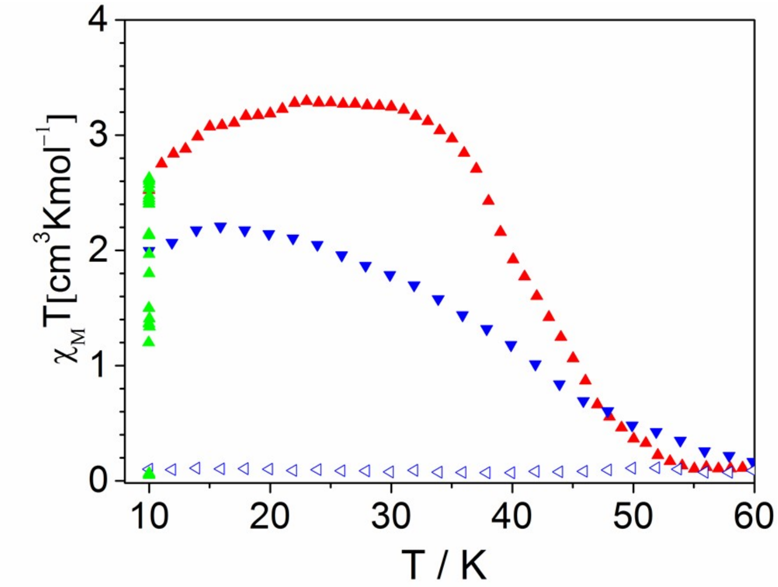

Figure S2. Changes of $\chi_M T$ upon continuous laser light irradiation (532 nm, 5 mW) for **1** during heating (red triangles, 0.3 K/min) and cooling (filled blue triangles, 0.3 K/min). Green triangles denotes increase of $\chi_M T$ during laser light irradiation (532 nm, 5 mW) after initial cooling to 10 K (1 K/min, blue empty triangles). Applied magnetic field 1 T.

HS*→LS relaxation in 1

Relaxation measurements for **1** were made in the 12-33 K range. The sample was irradiated until magnetization reached saturation, then the temperature was raised to the chosen value, the laser was turned off and data collection was started immediately. Data collection continued until relaxation when the magnetization values no longer changed. We found that relaxation starts practically immediately. At 33 K, after about 20 minutes half of the HS Fe(II) ions converts into the LS form. The deviation of the relaxation curves from the single exponential type dependence is noticeable. After relaxation starts, the process accelerates, and the sigmoidal character of the process is noted.[11] The measurement data were fitted according to the sigmoidal model to determine the kinetics of relaxation (see below).

$$d\gamma_{HS}/dt = - k^*_{HL}\,\gamma_{HS}$$

$$k^*_{HL}\,(T, \gamma_{HS}) = k_{HL}(T)\,\exp[\alpha(T)(1 - \gamma_{HS})$$

$$k_{HL}(T) = k_{\infty}\,\exp(E_a/k_BT)$$

Figure S3 shows the experimental data as well as the results of least-squares fits of experimental data $\gamma_{HS}(t)$ (γ_{HS} – molar ratio of HS form, t – time).

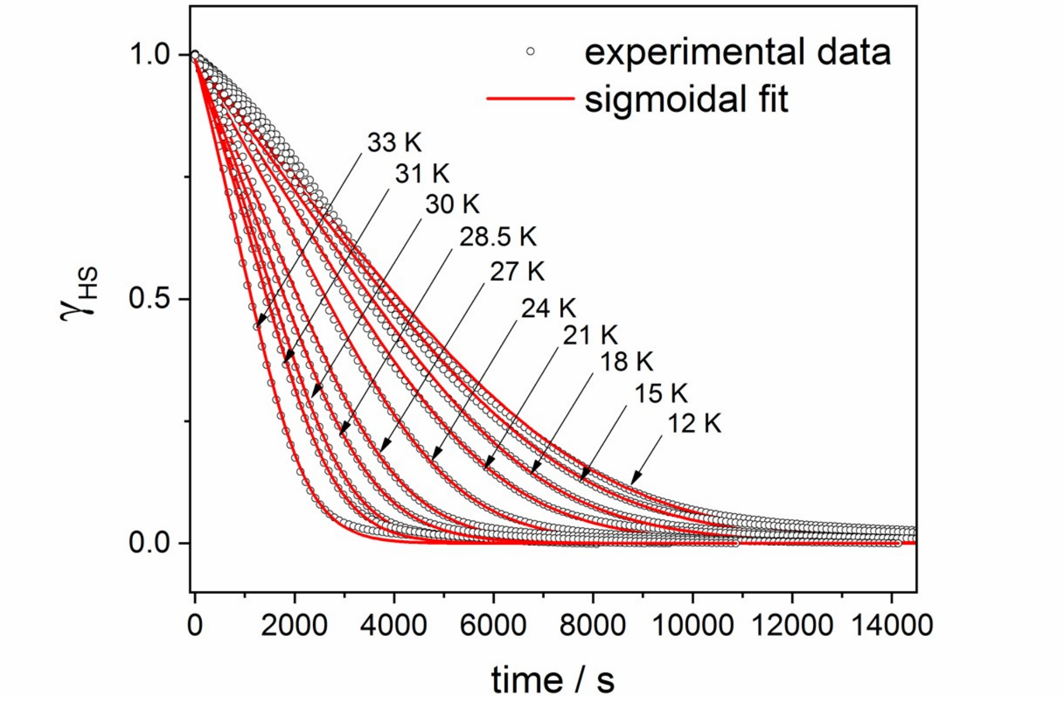

Figure S3. Relaxation kinetics of the γ_{HS} (HS fraction) versus t (time) at different temperatures for **1**.

Derived relaxation rate $k_{HL}(T)$ dependence (Fig. S2) indicates that at the highest temperatures we could measure, the relaxation process is thermally activated. Below 25 K, the thermally activated process clearly slows down and a temperature-independent process begins to dominate, which is in agreement with the tunneling mechanism being independent of temperature. The relaxation rate constant for the temperature-independent region $k_{HL}(T\rightarrow 0)$ can be estimated to be $1{\cdot}10^{-4}$ s^{-1}. For the thermally activated region (temperature range 27-33 K), the relaxation parameters were determined

by a linear fit of ln($k_{HL}(T)$) versus 1/T (Fig. S4). The derived values of the preexponential factor were k_{∞} = 7.3·10^{-3} s^{-1} and the activation energy E_a = 140 cm^{-1}.

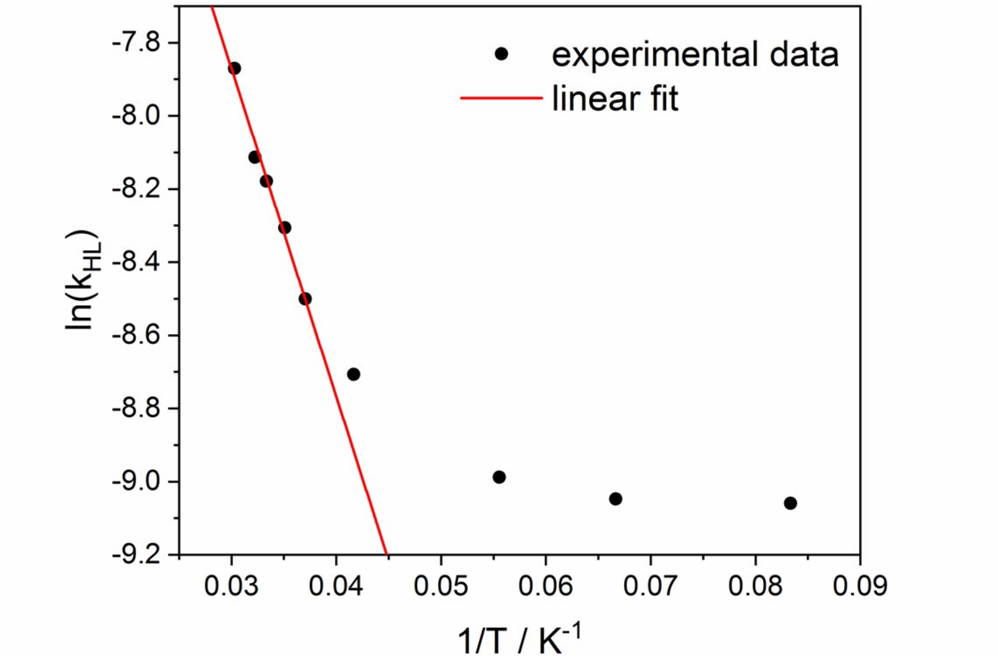

Figure S4. Arrhenius plot of ln(k_{HL}) versus 1/T for **1**.

From our data we were unable to reliably estimate the self-acceleration factor α(T) and the additional activation energy E_a*.[11] The temperature dependence of α is shown in Fig. S5.

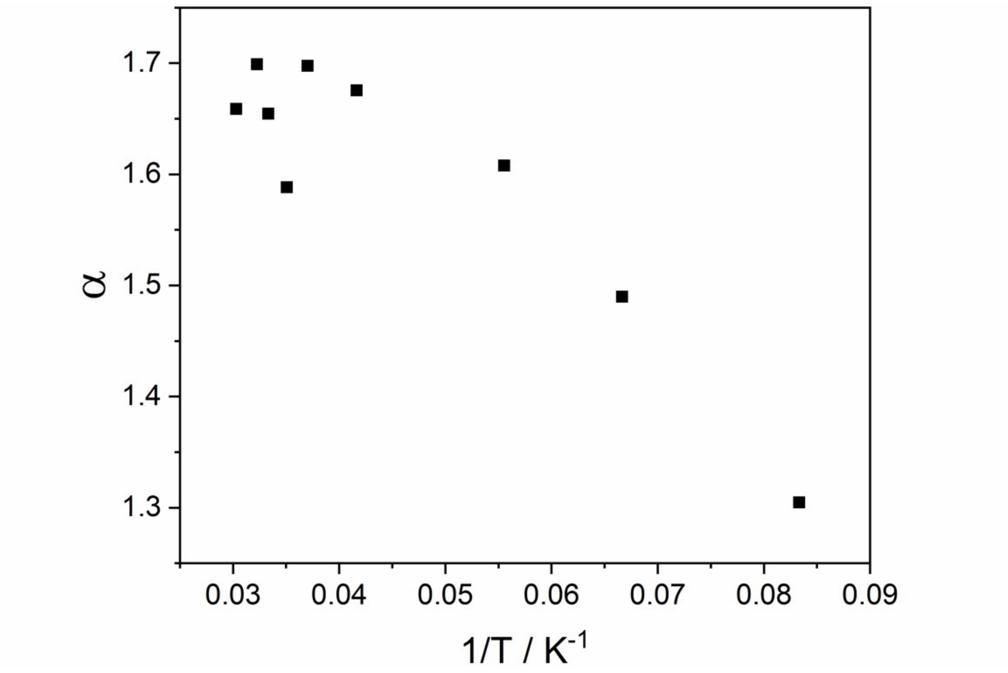

Figure S5. α acceleration factor versus 1/T for HS*$\rightarrow$LS relaxation in **1**.

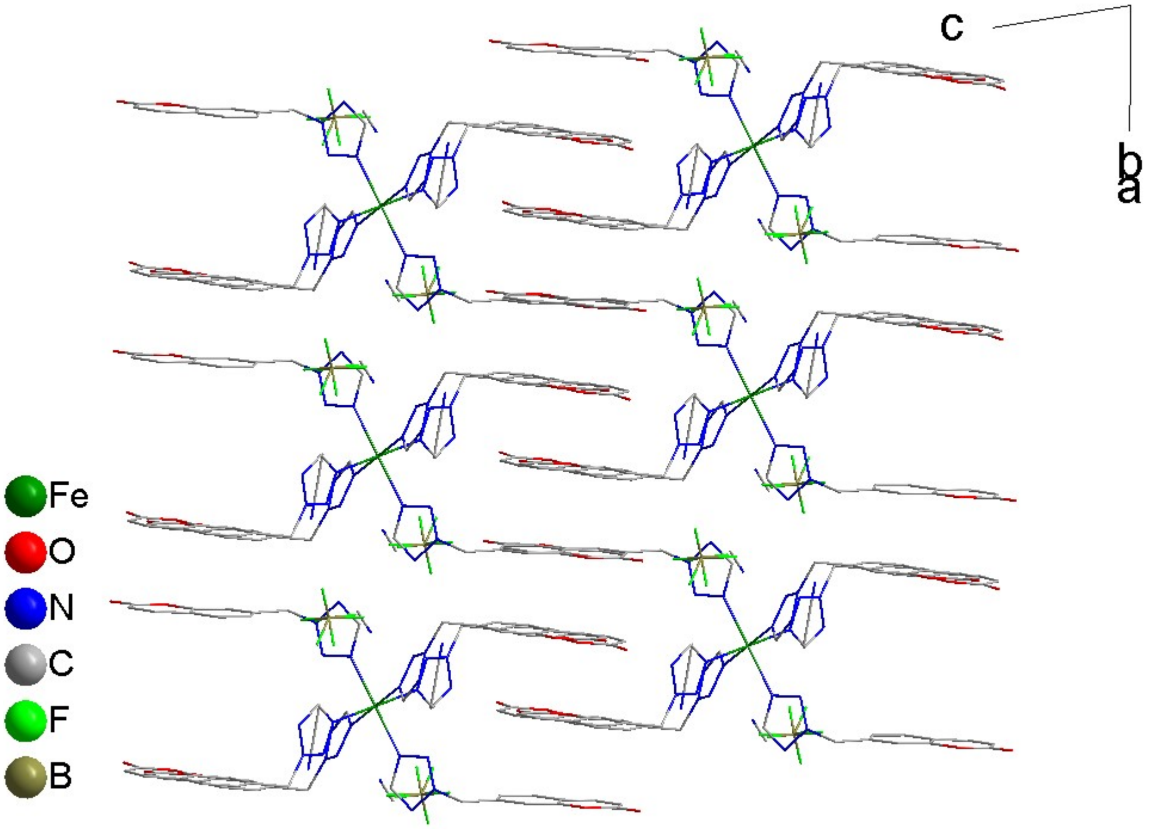

a)

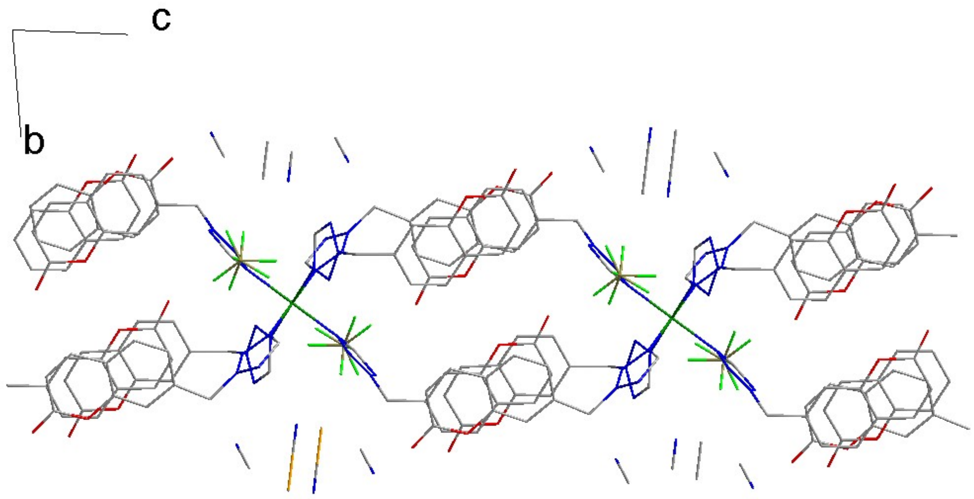

b)

Figure S6. Crystal packing showing pillared arrangement in **1**. Hydrogen atoms were omitted for clarity.

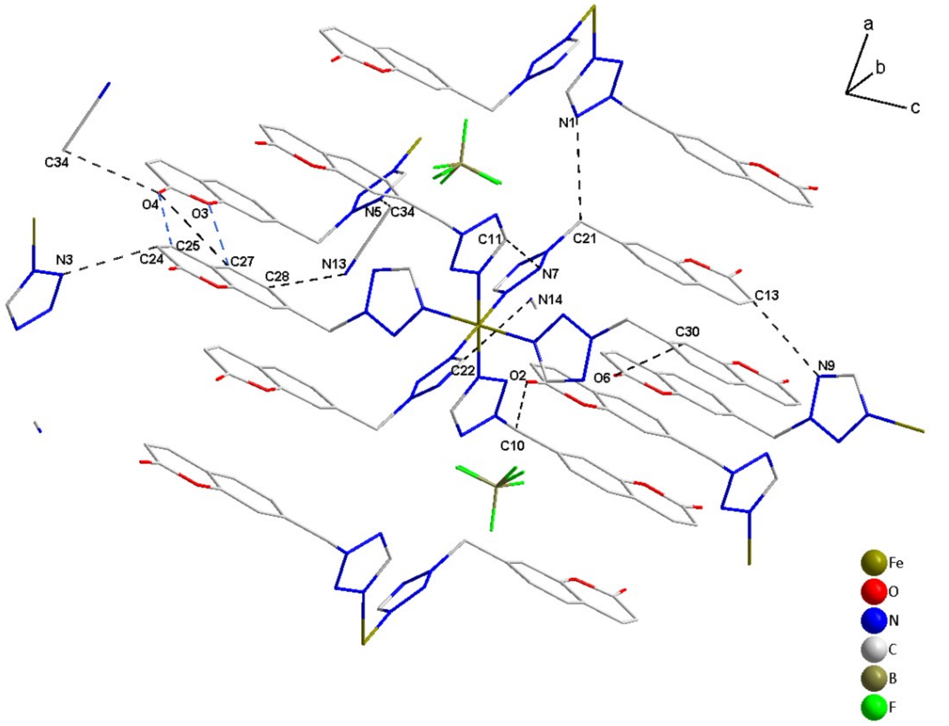

a)

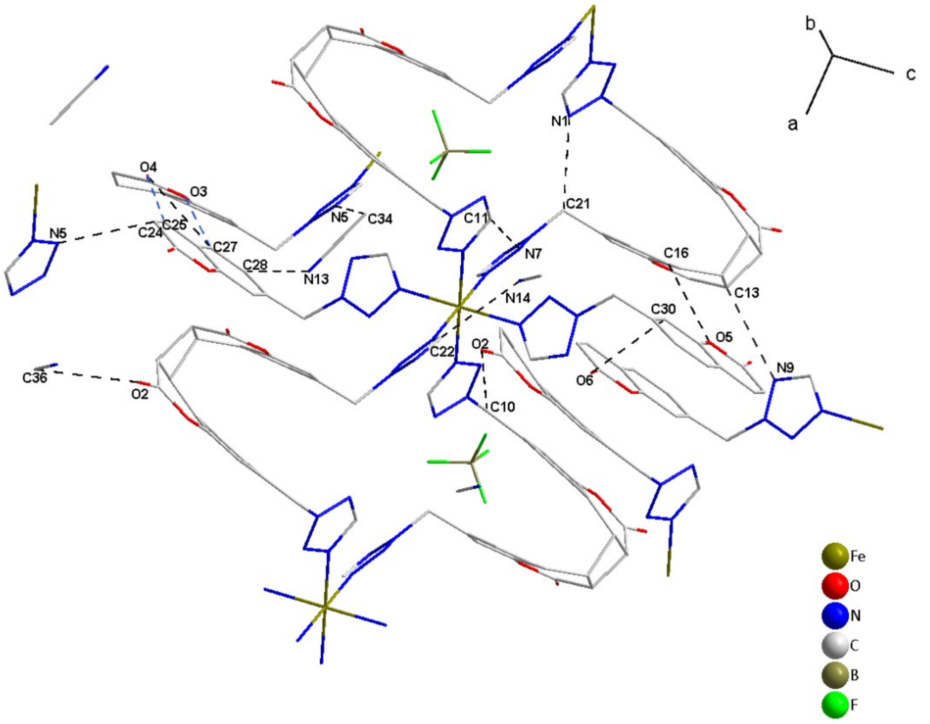

b)

Figure S7. C-H···N and C-H···O intermolecular contacts in **1** (**a**) and **2** (**b**). Black dotted lines denote contacts existing at 250 K and 80 K. Blue dotted lines show contacts presented only at 80 K. Contacts involving tetrafluoroborate anions are not depicted (see Table S3). Hydrogen atoms were omitted for clarity.

FTIR microscopy studies

FTIR monitoring of the photoconversion revealed changes primarily in the range of 1250-1750 cm^{-1} (Fig. 5b, Fig. S8a). In addition to the ν(C=O) band which is present in the primary sample **1** at 1740 cm^{-1}, a new intense band at 1750 cm^{-1}appears in the photoconverted sample **2.** Novel bands also occur at 1590, 1250 and 1220 cm^{-1}. DFT modelling of a high-spin mononuclear model and a pentanuclear chain model with dimerized ligand confirms the increase in intensity of the bands predicted around 1700 cm^{-1} involving the carbonyl group, as well as the appearance of new bands in the 1000-1260 cm^{-1} region. The band at about 1260 cm^{-1} for the pentanuclear HS model reveal a well-defined cyclobutane C-H stretching vibration. The calculated spectra for the model complexes (Fig S24) and the movies depicting characteristic modes for both complexes, as well as for monomeric and dimeric ligands, are provided in the Supplementary Materials. It is worth noticing that bands do not completely disappear after exhaustive exposure of the crystal to irradiation, which is consistent with the crystal structure of **1,** showing that two out of six ligand molecules cannot take part in photocyclization.

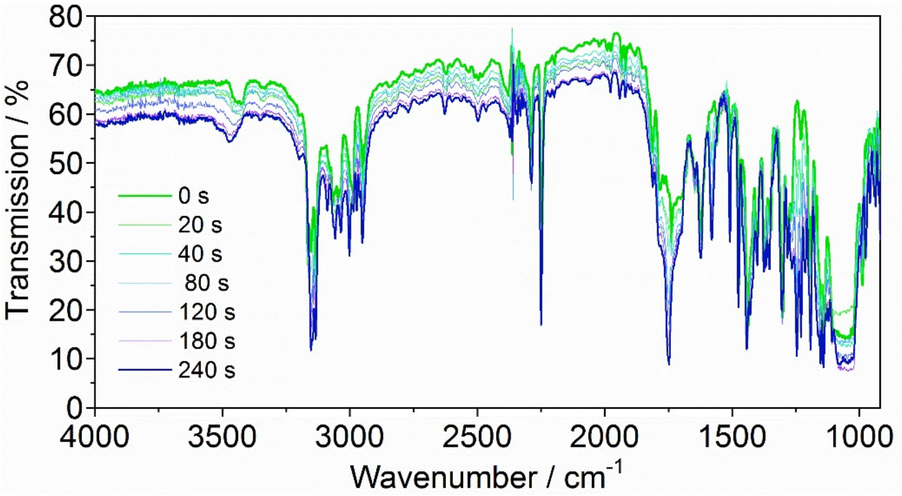

a)

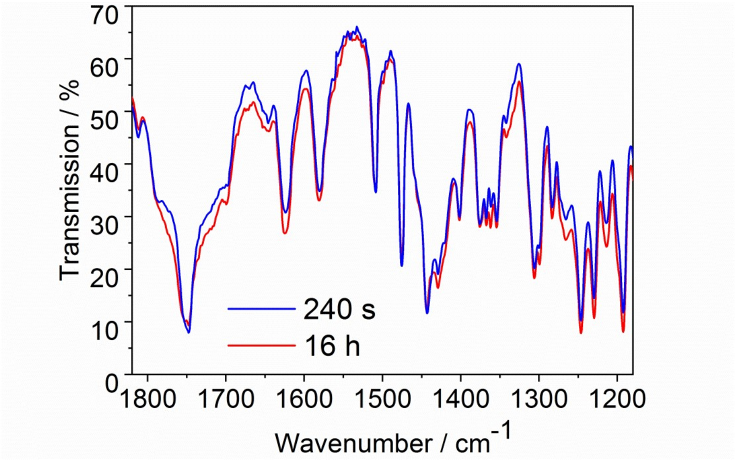

b)

Figure S8. Spatio-temporal FTIR microscopy spectra showing time evolution of spectra under irradiation (365 nm) (**a**). A comparison of spectra after 240 s and 16 hours of continuous irradiation (365 nm) (**b**).

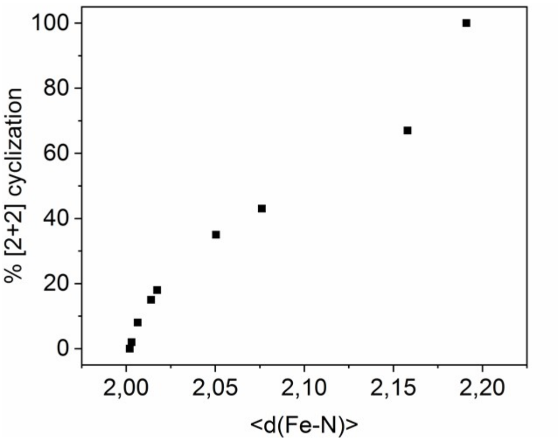

a)

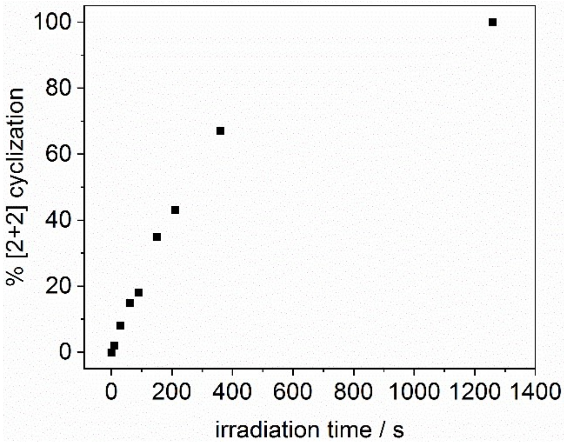

b)

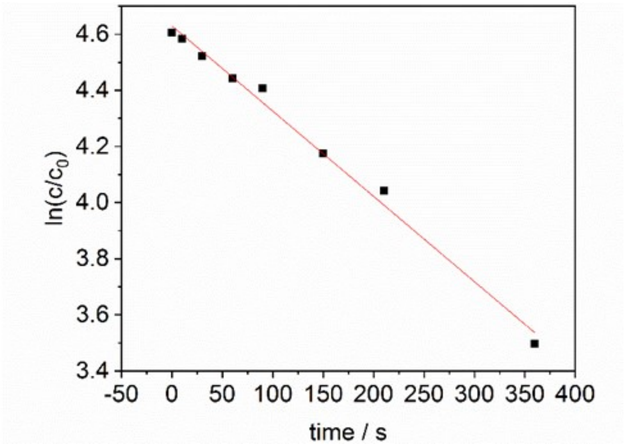

c)

Figure S9. Time dependence of [2+2] photocyclization (**a**) and change of average Fe-N distance (**b**) during single crystal irradiation (365 nm) under X-ray diffraction monitoring. Plot of $\ln(c/c_0)$ versus time (t) presented on (**c**) indicates first order behavior. Fitting $\ln(c/c_0)$ vs time dependence (red line represent best fitting) was carried out according to $\ln(c/c_0) = -kt$ equation (where k – reaction rate constant, c/c_0 represents the fraction of ligand converted to dimeric form). $k = 3.0(2)\cdot10^{-3}\ s^{-1}$.

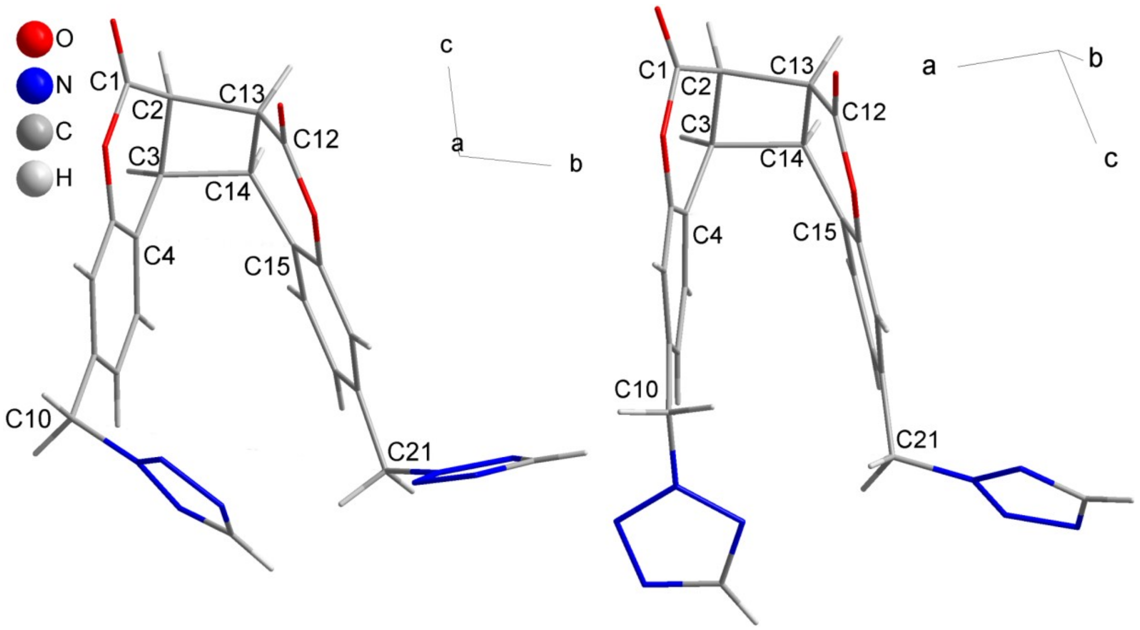

	L^{2+2}	L^2
C4-C15 / Å	2.943(2)	2.841(2)
C10-C21 / Å	6.281(3)	4.601(2)
C4-C3-C14-C15 / °	23.8(2)	17.5(1)
C4-C3-C14-C13 / °	-94.7(1)	-100.03(9)
C15-C14-C13-C12 / °	-20.9(2)	-16.4(1)
C15-C14-C13-C2 / °	-137.5(1)	-131.3(1)
C1-C2-C3-C4 / °	-23.5(2)	-20.8(1)
C1-C2-C3-C14 / °	-138.8(1)	-134.2(1)
C12-C13-C2-C3 / °	-100.2(1)	-105.47(9)
C12-C13-C2-C1 / °	22.8(2)	16.1(1)

Figure S10. A comparison of selected distances and torsion angles of cyclobutane derivatives in crystal structures of free cyclobutane derivative (**L^{2+2}**, left) and in the crystal structure of **2** (**L^2**, right) in 100 K.

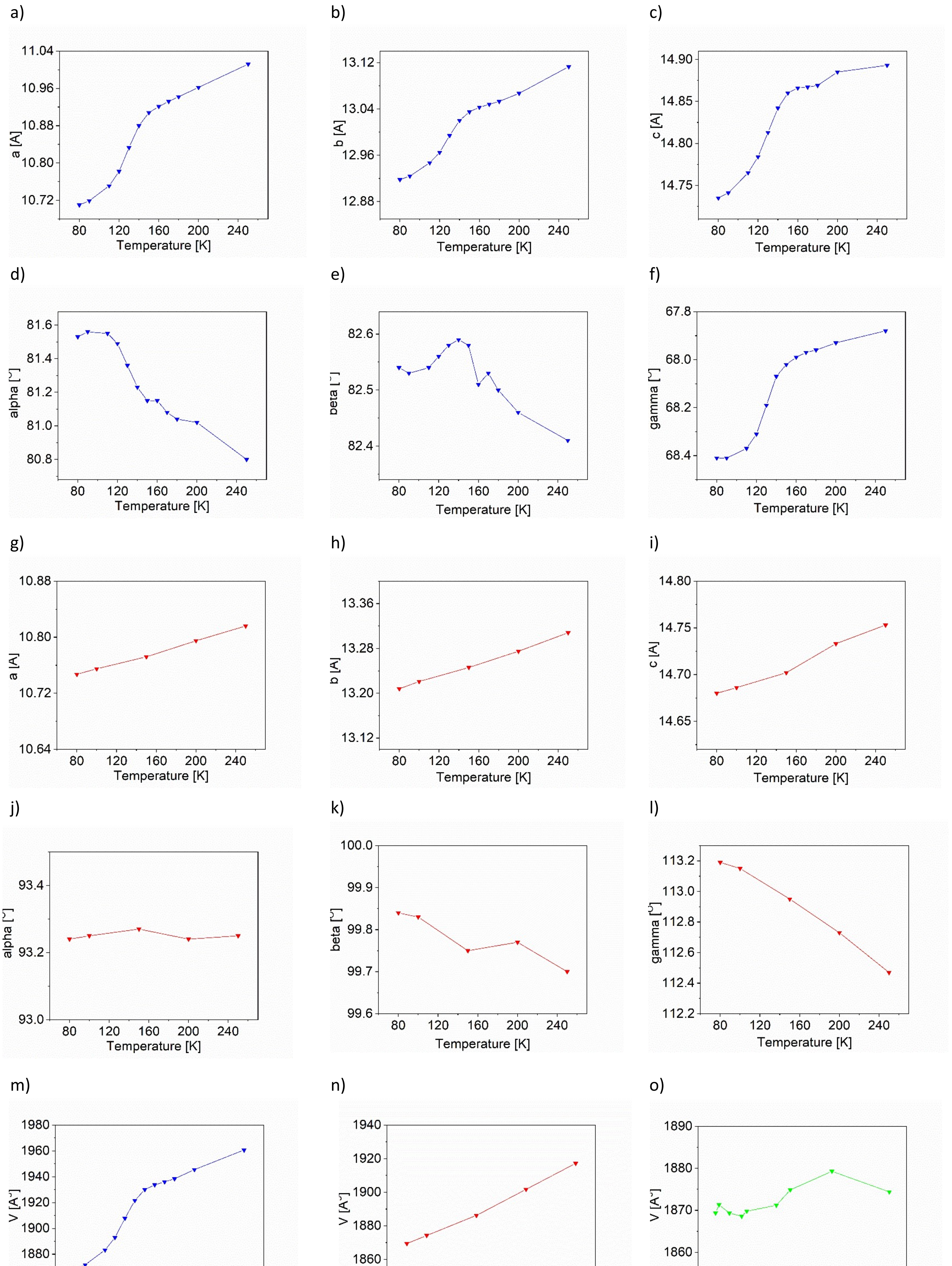

a)
b)
c)
11.04
10.96
10.88
10.80
10.72
a [A]
80
120
160
200
240
Temperature [K]
13.12
13.04
12.96
12.88
b [A]
14.90
14.85
14.80
14.75
c [A]
d)
e)
f)
81.6
81.4
81.2
81.0
80.8
alpha [°]
82.6
82.5
82.4
beta [°]
67.8
68.0
68.2
68.4
gamma [°]
g)
h)
i)
10.88
10.80
10.72
10.64
13.36
13.28
13.20
13.12
14.80
14.75
14.70
14.65
j)
k)
l)
93.4
93.2
93.0
100.0
99.9
99.8
99.7
99.6
113.2
113.0
112.8
112.6
112.4
112.2
m)
n)
o)
1980
1960
1940
1920
1900
1880
V [A³]
1940
1920
1900
1880
1860
1890
1880
1870
1860

p)

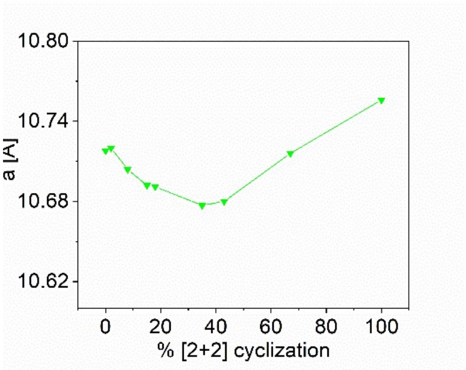

r)

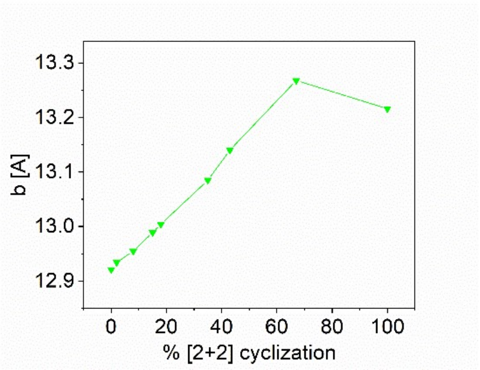

s)

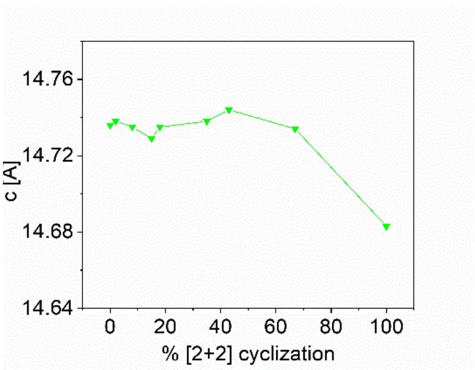

t)

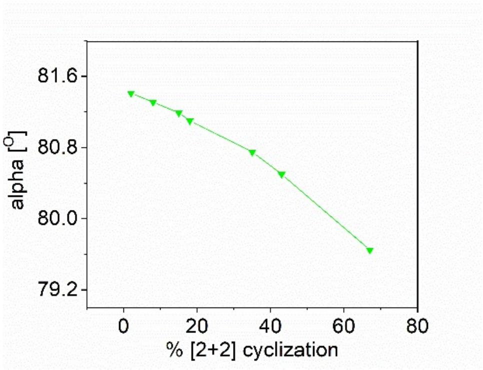

u)

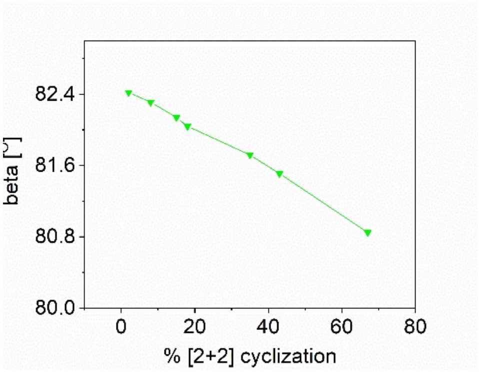

w)

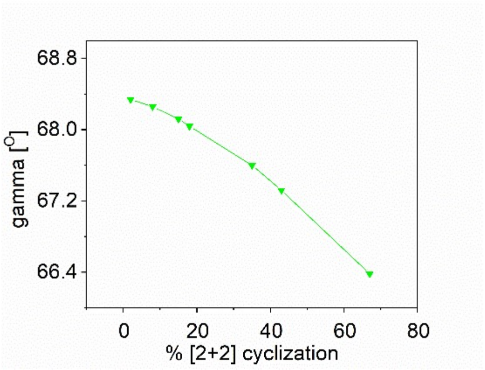

Figure S11. Temperature dependence of lattice parameters for **1** (**a-f**) and **2** (**g-l**). Temperature dependence of cell volume for **1** (**m**) and **2** (**n**). Dependence of cell volume (**o**) and lattice parameters (**p-w**) *vs* degree of [2+2] photocyclization.

Mössbauer spectroscopy studies

Mössbauer spectroscopy studies were carried out for **1** enriched with the ^{57}Fe isotope to approximately 8%. At 200 K, the spectrum consists of one quadrupole doublet with an isomer shift δ = 1.13 mms^{-1} and a quadrupole splitting ΔE_Q = 2.50 mms^{-1}, which is characteristic for the high spin form (HS1) of iron(II) (Fig. S12a).

Lowering the temperature below 150 K involves an appearance of a single line attributed to the low spin form (LS1). At 60 K, there is solely a low-spin component in the spectrum (δ = 0.57 mm^{-1}, ΔE_Q = 0.22 mms^{-1}, Fig. 12b). A measurement performed after continuous irradiation (532 nm) of **1** at 25 K indicates the appearance of a metastable high spin form HS1* with δ = 1.19 mms^{-1} and ΔE_Q = 2.83 mms^{-1} (Fig. S12d). In this experiment, approximately 58% of Fe(II) was converted to the metastable HS1* form which remains consistent with the results of the photomagnetic LITH experiment. After switching the light off HS1* $\rightarrow$ LS1 relaxation occurs. Mössbauer spectrum for compound **2** is presented on (c).

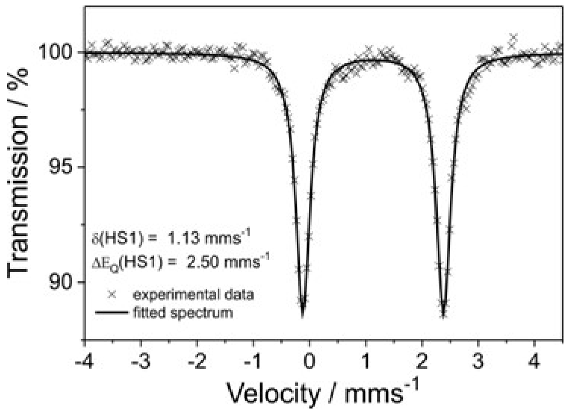

a)

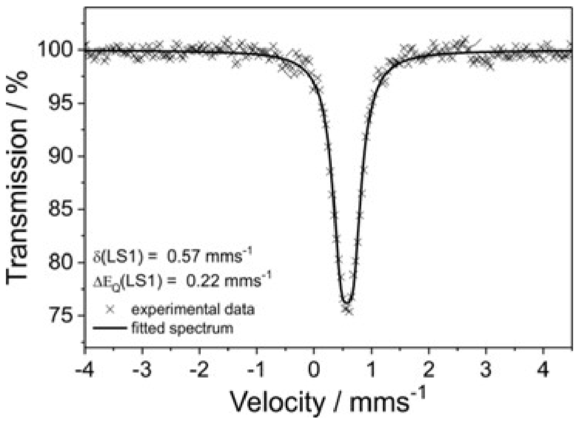

b)

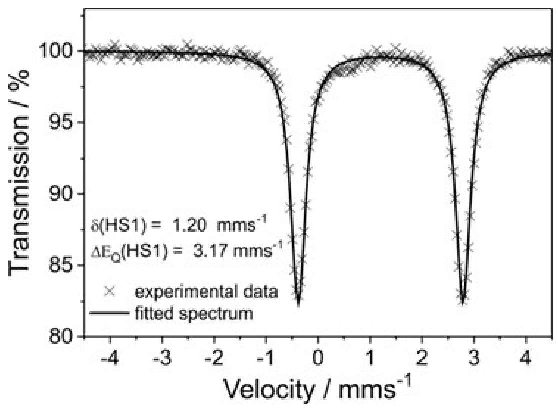

c)

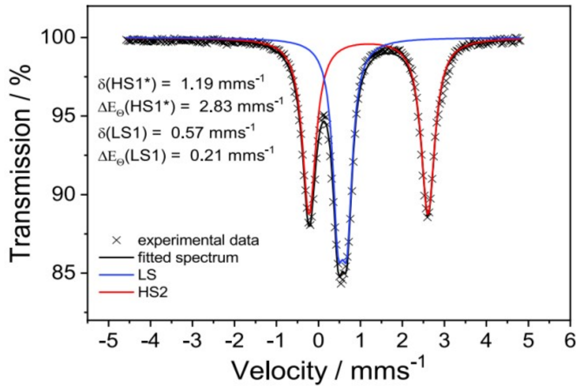

d)

Figure S12. Mössbauer spectra (cooling mode) for **1** at 200 K (**a**) and 60 K (**b**) as well as for **2** at 60 K (**c**) together with isomer shift (δ) and quadrupole splitting (ΔE_Q) parameters (inset). The line widths at half maximum as obtained from the analysis were 0.31, 0.37 and 0.36 mms^{-1} for (**a**), (**b**) and (**c**). Figure (**d**) presents Mössbauer spectrum of **1** recorded under continuous light irradiation (532 nm) at 24 K.

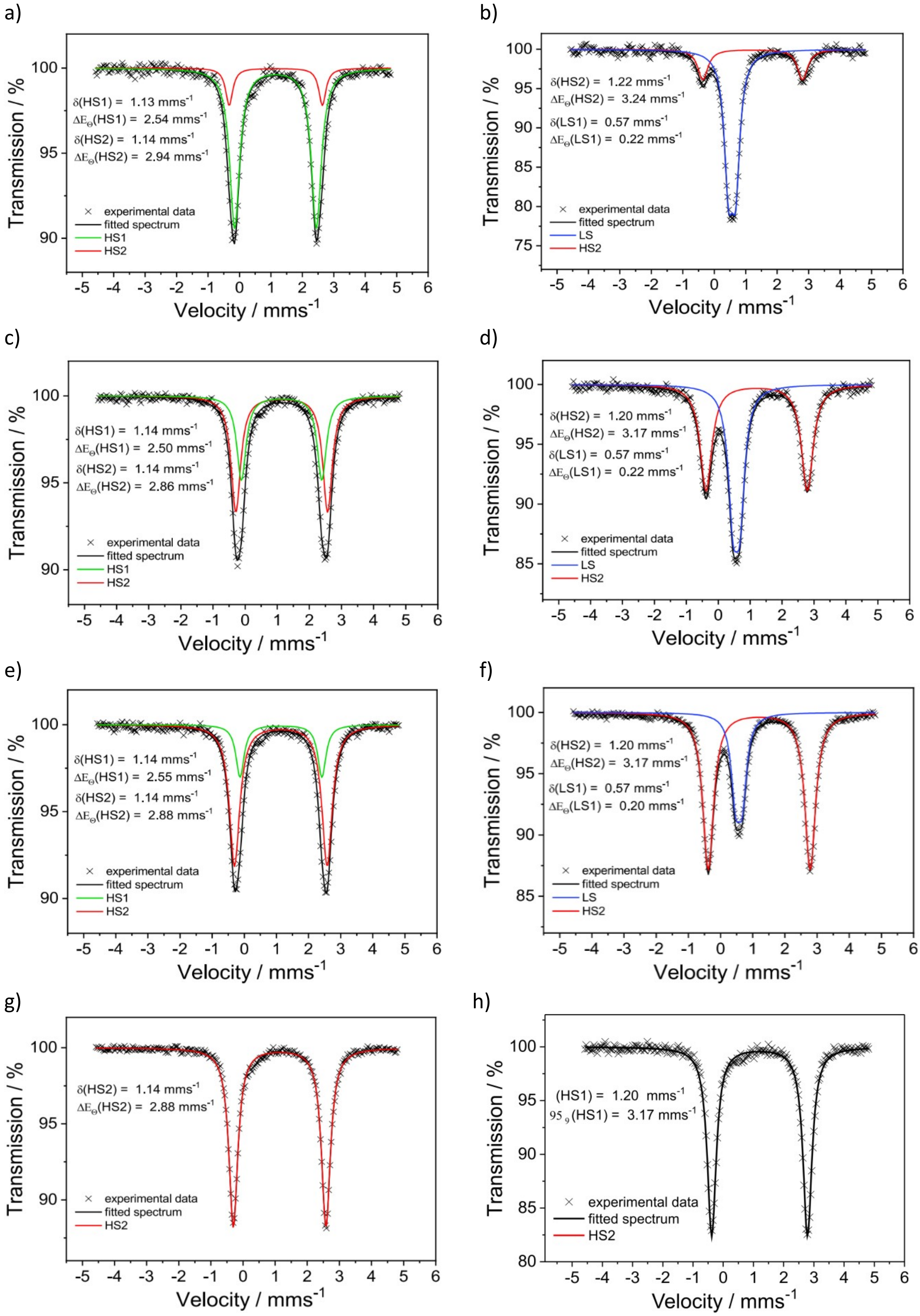

Figure S13. Mössbauer spectra of **2** containing 23 (**a, b**), 54 (**c, d**), 77 (**e, f**) and 100% (**g, h**) of HS2 component at 200 (left column) and 60 K (right column).

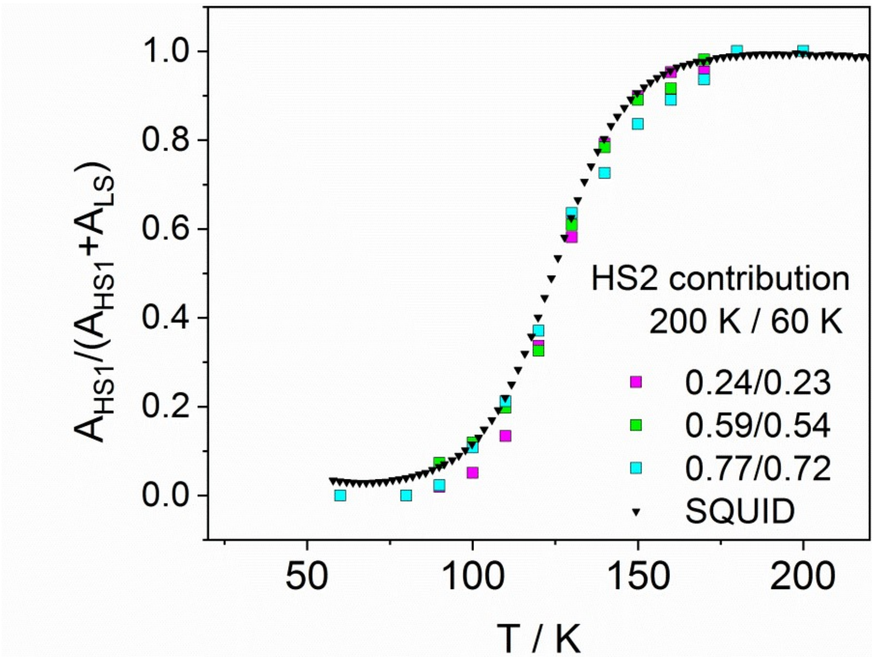

Figure S14. HS1 → LS dependences derived from Mössbauer spectroscopy according to $A_{HS1}/(A_{HS1}+A_{LS})$ relationship for different contributions of HS2 component. For comparison molar fraction of HS form γ_{HS} determined from magnetometry (SQUID, black triangles, temperature scan rate 1 K/min, magnetic field 1 T) was added.

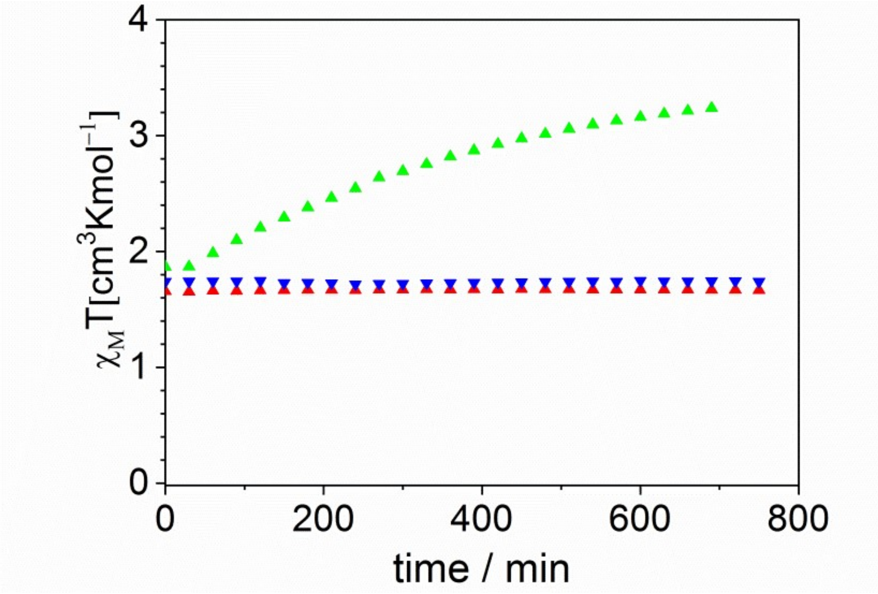

a)

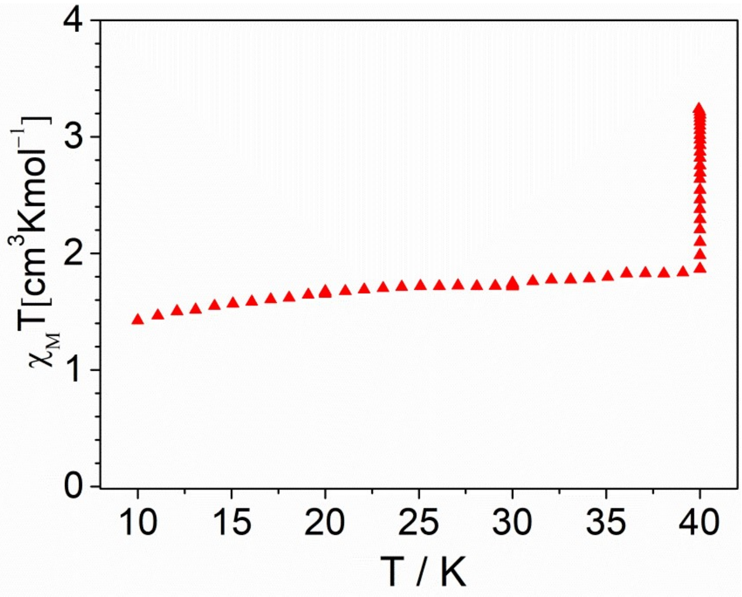

b)

Figure S15. Time evolution of $\chi_M T$ value in temperature 20, 30 and 40 K (**a**). Applied magnetic field 1 T. After irradiation of HS2 to LS2 the temperature was elevated (1 K/min) and sample was left above 10 h at 20 K (**a**, red triangles). Then the temperature was raised (1 K/min) to 30 K and again the sample was left in this temperature (**a**, blue triangles). Finally the temperature was elevated (1 K/min) to 40 K (**a**, green triangles). On (**b**) there is presented the $\chi_M T$ value evolution in the function of temperature during the experiment.

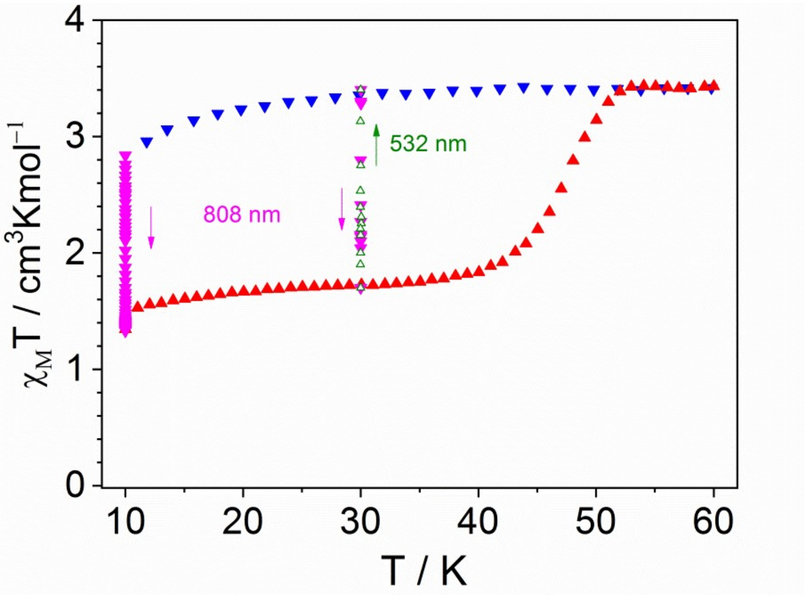

Figure S16. Bidirectional HS→LS and LS→HS switching in **2**. Hidden hysteresis has been included for reference (cooling were marked by blue and red color, respectively; dependences recorded at temperature scan speed 0.3 K/min; applied magnetic field 1 T).

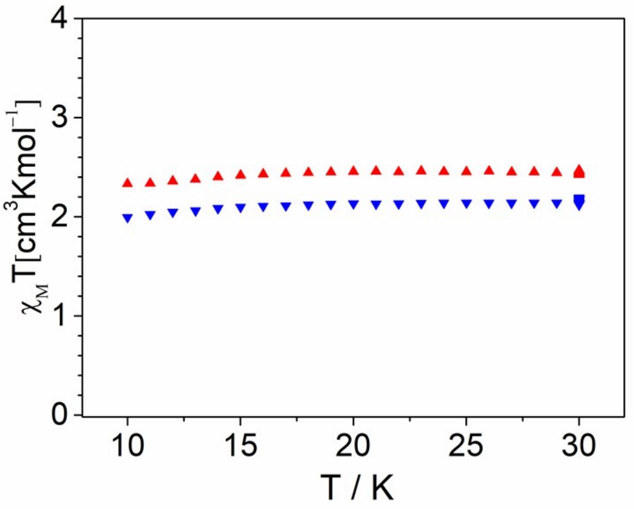

a)

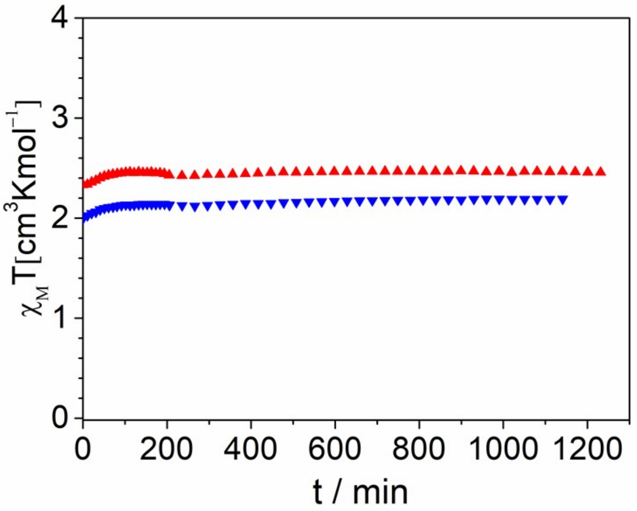

b)

Figure S17. Temperature (**a**) and time (**b**) evolution of $\chi_M T$ value in temperature 30 K for two different partial HS2 $\rightarrow$ LS2 switching. After irradiation of **2** resulting in partial switching of HS2 to LS2 the temperature was elevated (0.3 K/min) and sample was left at 30 K for about 20 h. Applied magnetic field 1 T.

DFT Modelling of the molecules and the stress therein.

Geometry of all model molecules was optimized followed by the normal mode analysis. The spin isomers of the mononuclear **1** were modelled for the +2 cationic species with the starting geometry obtained from the X-ray structures. The obtained optimized structure are shown in Figure S18, the pdb files are attached as Supplementary Materials. The obtained Fe-N bond lengths lie in the range 1.990-2.012 Å and 2.157-2.214 Å for the LS and HS state, respectively, being in reasonable agreement with those found experimentally. Expectedly, the orientation and conformation of the ligands resulting form the crystal packing could not be reproduced by the modelling for the single molecule.

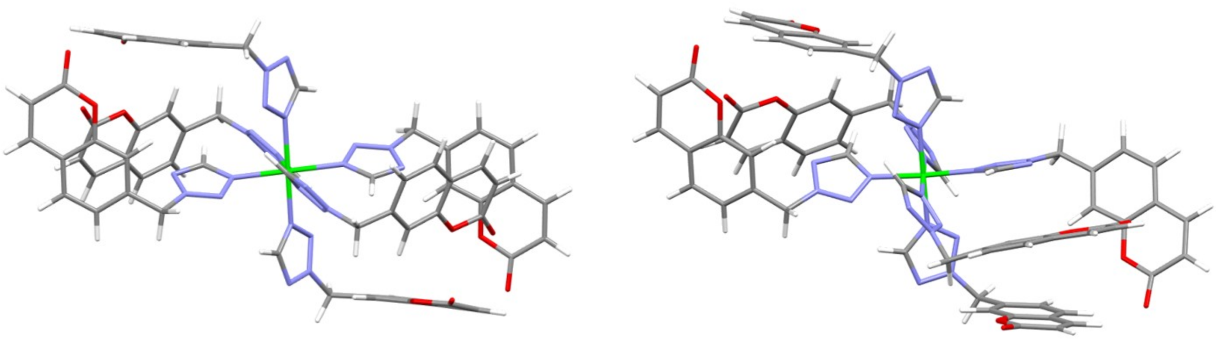

Figure S18. Optimized structures of the HS (right) and LS (left) dicationic models of **1**.

The DFT modelling for the HS and LS isomers of **2** were performed for the pentanuclear models shown in Fig. S19. Again the starting from the X-ray data for HS spin isomer. Eight BF_4^- anions were included in the model molecule resulting in the in the net charge of +2. Three inner Fe-centres are coordinated with four dimerized ligands and two monomeric ones. The two terminal atoms of the pentanuclear chain are coordinated with two dimerized and two monomeric ligands accompanied with two methyl-tetrazole closing the structure. The obtained Fe-N distances for the HS isomer lie in the range 2.132-2.205 Å, again being in fair agreement with the X-ray data and close to that calculated for the high-spin isomer of **1**. for the central Fe coordination core.

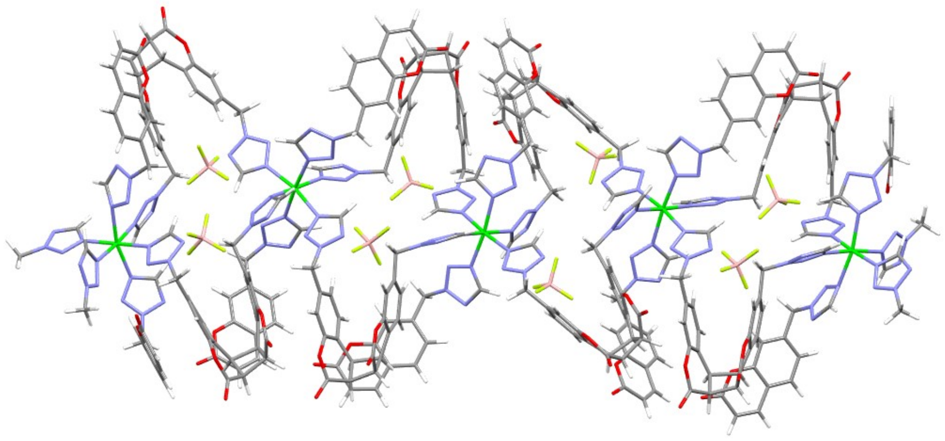

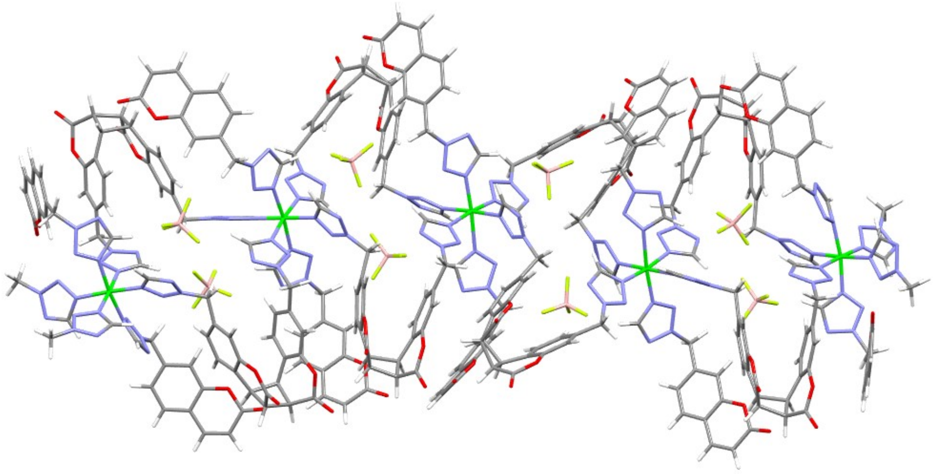

Figure S19. Optimized structures of the HS (top) and LS (bottom) pentanuclear models of **2**.

Note that the optimized structure of the HS model of **2** deviates form the linear structure determined on the basis of the X-ray diffraction. The similar distortion was obtained for the LS model of **2**. The calculated Fe-N distances for this model point towards a quite significant strain. For the central Fe-N core the average Fe-N bond distance is 2.092 Å, nearly 0.1 Å despite the results of the normal vibration analysis, showing no imaginary frequencies. Furthermore, the optimization of the geometry of the central mononuclear unit cut from the optimized pentanuclear model of low-spin **2** (see Fig. S20) brings about the decrease of the electronic energy of ca. 220 kJ/mol compared to initial geometry of yet the average Fe-N bond length is even slightly increased to 2.097 Å. That implies that interligand strain entirely dominates the radial strain due to elongation of the low-spin Fe-N bonds. Hence the coordination of the photodimerized ligand under study leads to a very high-stabilisation of the high-state due to interligand repulsion.

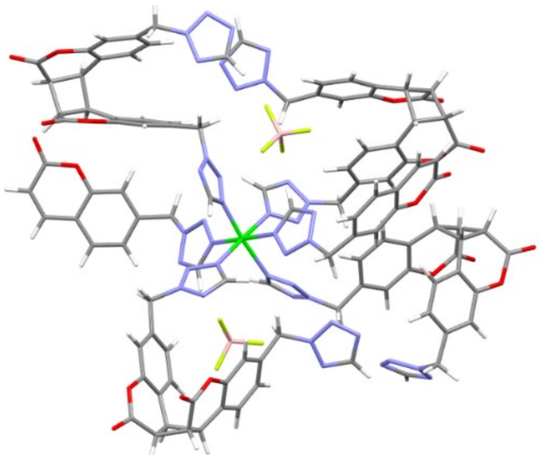

Figure S20. Optimized structure of the central unit of the LS pentanuclear models of **2**.

In the final step the obtained structures were used to build up the starting geometries of the analogues of **2** in which in the dimerized ligands have the CH_2CH_2 between the coumarine and tetrazole rings instead of the methyl one. The monocoordinating ligands of monomeric coumarinetetrazole were kept as in **2**. The optimized structures of these system named **2b** are shown in Fig. S21. In fact, this increase of the flexibility of the bridging dimeric ligands releases the distortion of the metal-ligand bonds in the LS state - with the Fe-N distances in the range of 1.989-2.033 Å. Interestingly, the corresponding structure of the high-spin pentanuclear complexes reveals the Fe-N bond slightly shorter than for **2**, the ones for the central Fe-N core lying in the range 2.15-2.17 Å.
In order to asses the stress due to coordination of the dimerized ligands we first looked at the geometry of the optimized free ligand (see the pdb file attached). As there are eight dimerized ligands in the pentanuclear model of **2** we may compare the structure of the free ligand with eight of the coordinated ones. The inspection of the optimized geometries reveals that the strain upon coordination is equally distributed into several angles and distances so there is no one single geometrical parameter that is particularly affected. Yet the calculation of the electronic energies of the obtained structures reveals a significant increase of the electronic energy on going from the free ligand to these obtained for the pentanuclear model of HS2. The energy increase is on average 34 +- 4 kJ/mol with the increase varying from 29 to 43 kJ/mol. Thus, the strain induced by coordination brings about a stress of few tens of kJ/mol.

In the next step we compared the electronic energies of the dimeric ligands in the HS structure of the model with that of the corresponding LS one. The obtained average change on going form the LS to HS structure is only 2 +- 7 kJ/mol with the values varying from +14 to -7 kJ/mol. Thus the electronic energy difference between HS and LS pentanuclear modes to be -5 kJ/mol per iron centre compared to +14 kJ/mol for the monomeric is not due to increase of the strain of the dimeric ligands upon shortening of Fe-N bonds on going from the HS to the LS state. The possible explanation is that the strain in the coordinated ligand can not be further increased further. The remaining possibility is the increase of the Fe-Fe distance on HS to LS switching brings about the repulsive ligand interactions already discussed above for the cut geometry of the central coordination unit. In order to estimate this factor we calculated the electronic energy for the dimeric ligands assemblies obtained from the optimized structures of the pentanuclear model. In fact the calculation for the systems shown in Fig. S22, involving eight dimeric ligands yields the electronic energy difference of 110 kJ/mol in favour of

the assembly corresponding to the HS structure of the model of **2.** As stated in the main text the elasticity introduced in the model **2b** leads to further stabilisation of the HS strain.

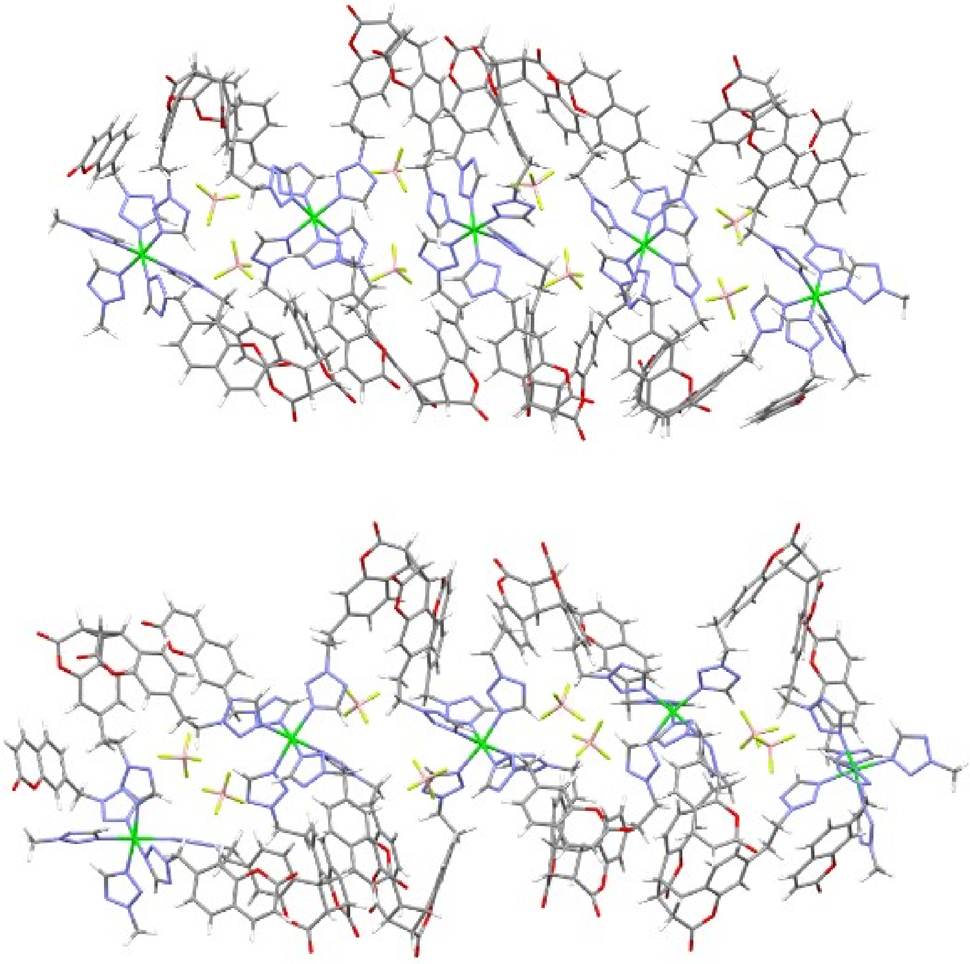

Figure S21. Optimized structures of the HS (bottom) and LS (top) pentanuclear models of **2b**.

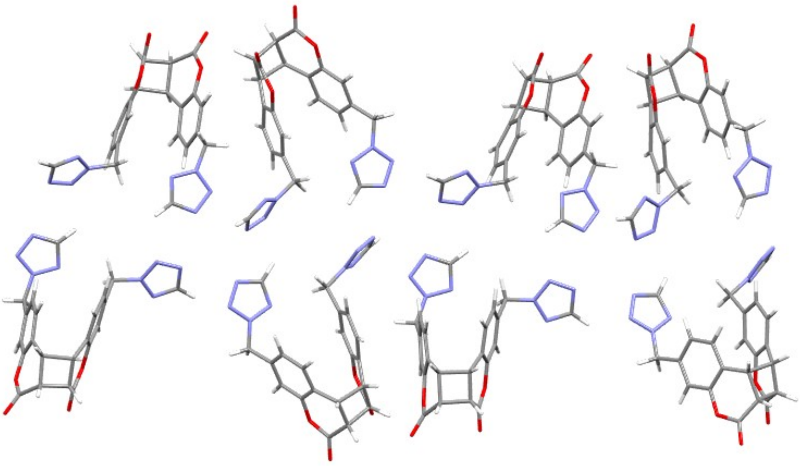

Figure S22. Dimeric ligand assembly used for the calculation of the interligand repulsion in models of **2**.

The IR spectra of both high-spin spin isomers of **2** were calculated using the optimized pentanuclear model. For the high-spin isomer of **1** two model were used. First model involved the optimized monomeric cationic unit, the second one involved the nine molecules forming the 2D sheet, cut off from the X-ray structure (see Fig. S23) in which only the central one was optimized and for which the normal mode analysis was performed. The procedure corresponded to that recently described in ref. 12. To simplify the calculations all high-spin Fe(II) centres were replaced with Zn(II) known to be a good structural model for HS Fe(II). The comparison of the experimental and calculated spectra in the area of the bands diagnostic for the dimerization are shown in. Fig. S24. The movies showing the diagnostic vibrations are given separately as ESI.

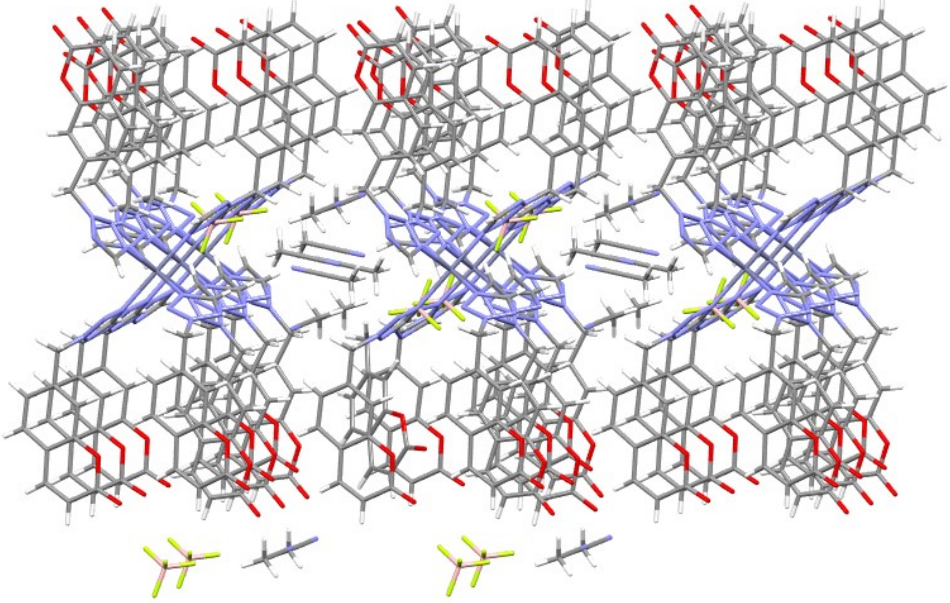

Figure S23. The molecular assembly used for the calculation of IR spectra of high-spin of **1** involving the intermolecular interactions.

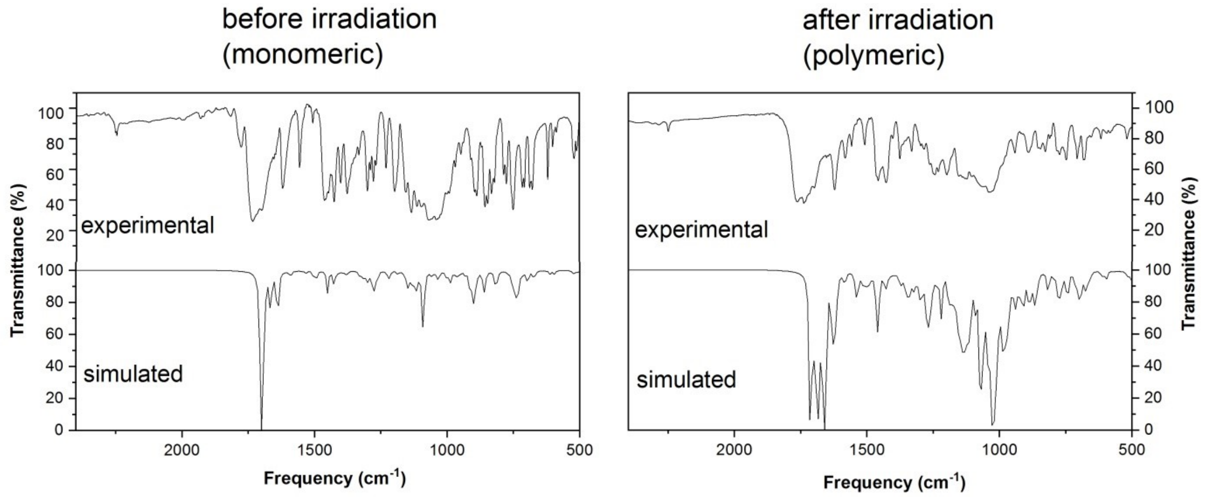

Figure S24. Experimental and simulated spectra for high spin forms of **1** (monomeric) and **2** (polymeric) species.

References

1. R. Bronisz, *Inorg. Chim. Acta*, 2002, **340**, 215.
2. A. B. Gaspar, G. Molnár, A. Rotaru and H. J. Shepherd, *C. R. Chimie* 2018, **21**, 1095.
3. CrysAlisPro 1.171.42.49, Rigaku Oxford Diffraction, 2022.
4. a) G. M. Sheldrick, *Acta Crystallogr.*, *Sect. C: Struct. Chem*., 2015, **71**, 3; b) , O.V. Dolomanov, L.J. Bourhis, R.J. Gildea, J.A.K Howard and H. Puschmann, *J. Appl. Cryst.* 2009, **42**, 339-341.
5. Gaussian 16, Revision A.03, M. J. Frisch, G. W. Trucks, H. B. Schlegel, G. E. Scuseria, M. A. Robb, J. R. Cheeseman, G. Scalmani, V. Barone, G. A. Petersson, H. Nakatsuji, X. Li, M. Caricato, A. V. Marenich, J. Bloino, B. G. Janesko, R. Gomperts, B. Mennucci, H. P. Hratchian, J. V. Ortiz, A. F. Izmaylov, J. L. Sonnenberg, D. Williams-Young, F. Ding, F. Lipparini, F. Egidi, J. Goings, B. Peng, A. Petrone, T. Henderson, D. Ranasinghe, V. G. Zakrzewski, J. Gao, N. Rega, G. Zheng, W. Liang, M. Hada, M. Ehara, K. Toyota, R. Fukuda, J. Hasegawa, M. Ishida, T. Nakajima, Y. Honda, O. Kitao, H. Nakai, T. Vreven, K. Throssell, J. A. Montgomery, Jr., J. E. Peralta, F. Ogliaro, M. J. Bearpark, J. J. Heyd, E. N. Brothers, K. N. Kudin, V. N. Staroverov, T. A. Keith, R. Kobayashi, J. Normand, K. Raghavachari, A. P. Rendell, J. C. Burant, S. S. Iyengar, J. Tomasi, M. Cossi, J. M. Millam, M. Klene, C. Adamo, R. Cammi, J. W. Ochterski, R. L. Martin, K. Morokuma, O. Farkas, B. Foresman, and D. J. Fox, Gaussian, Inc., Wallingford CT, 2016.
6. A. D. Becke *J. Chem. Phys., 1993,* **98**, 5648.
7. a) W.J. Stevens, H. Basch and M. Krauss, *J. Phys. Chem.* 1984, **81**, 6026; b) W.J. Stevens, M. Krauss, H. Basch and P.G. Jasien, *Can. J. Chem.,* 1992, **70**, 612; c) T.R. Cundari and W.J. Stevens, *J. Chem. Phys.,* 1993*,* **98**, 5555.
8. S. Grimme , J. Antony, S. Ehrlich and H. Krieg *J. Chem. Phys.,* 2010, **132**, 154104.
9. J. Zhou, Y. Luo, Q. Li, J. Shen, R. Wang, Y. Xu and X. Qian, *New J. Chem*., 2014, **38**, 2770.
10. J. N. Moorthy, P. Venkatakrishnan, G. Savitha and R. G. Weiss, *Photochem. Photobiol. Sci*., 2006, **5**, 903.
11. A. Hauser A, J. Jeftic, H. Romstedt, R. Hinek, H. Spiering, *Coord Chem Rev*, 1999, , **190–192**. 471-491.
12. J. A. Wolny, K. Gröpl, J. Kiehl, E. Rentschler and V. Schünemann, *Dalton Trans.,* 2024, **53**, 8391.
